# Giving AI agents a sense of control facilitates reinforcement learning in multitasking scenarios

**DOI:** 10.1371/journal.pone.0342305

**Published:** 2026-02-06

**Authors:** Annika Österdiekhoff, Nils Wendel Heinrich, Nele Russwinkel, Stefan Kopp

**Affiliations:** 1 Social Cognitive Systems, Faculty of Technology, CITEC, Bielefeld University, Bielefeld, Germany; 2 Institute of Information Systems (IFIS), Department of Computer Science and Engineering, Universität zu Lübeck, Lübeck, Germany; University of Zagreb Faculty of Electrical Engineering and Computing: Sveuciliste u Zagrebu Fakultet Elektrotehnike i Racunarstva, CROATIA

## Abstract

Having to control multiple tasks in parallel poses challenges for humans and artificial agents alike. In artificial intelligence, specific forms of reinforcement learning (RL), most notably hierarchical and model-based RL, have shown promising results in scenarios where tasks or skills need to be switched adaptively. However, RL agents still encounter difficulties when faced with serial multitasking that involves switching control between continuously running subtasks, such as changing the radio station while driving in traffic. Inspired by human cognitive processes, we hypothesize that maintaining a sense of control is a key mechanism facilitating such task-switching decisions. We propose a mathematical formulation of a situational sense of control that consists of two components: an evaluative indicator of the predictability of action outcomes and a predictive indicator of a need for control in individual subtasks. We integrate this model of a sense of control into a hierarchical RL agent and evaluate its performance in a *Collect Asteroids* game environment, in which one must alternate between navigating two spaceships to collect as many asteroids as possible. Comparing RL agents with and without a sense of control, as well as with human participants, shows that equipping RL agents with a sense of control results in significant performance improvements. Our findings indicate that agents equipped with a sense of control prioritize more complex tasks, exhibit increased switching behavior, and make switches at strategically optimal times, leading to superior overall performance. The incorporation of cognitive mechanisms, inspired by human behavior, into RL agents thus appears to yield considerable enhancements in performance when acting in complex and dynamic environments.

## Introduction

Recent advances in the field of Artificial Intelligence (AI) have enabled the implementation of autonomous systems such as cars, robots, or personal assistants that can act in uncertain or dynamic scenarios [1–3]. In particular, Reinforcement Learning (RL) provides a robust framework for identifying an optimal decision-making “policy” for an autonomous “agent” by selecting actions to interact with the environment and learning from resulting observations and rewards. The corresponding trial-and-error learning process derives an optimal policy that maximizes cumulative rewards [4]. This approach has achieved notable successes in environments such as board games like Go or Chess, and various Atari games [5–9]. However, despite these successes, RL agents encounter considerable challenges when having to manage multiple tasks concurrently within uncertain and dynamic environments [10]. Consider, for example, changing the radio station while driving in traffic. In these situations, which we encounter frequently in our normal environment, humans manage their perceptual, attentional, cognitive, and motor resources to direct control to single subtasks while keeping track of and switching to other subtasks in order to manage the overall multitasking situation successfully.

Multitasking refers to the capability of managing multiple tasks simultaneously to achieve an overall goal. It is extensively investigated by cognitive scientists researching task-switching decisions that give rise to *serial* multitasking in humans [11–13]. In contrast to *concurrent* multitasking, where individuals are required to address two tasks that are being executed simultaneously, serial multitasking involves transitioning between several tasks [14]. Different studies investigated *internally* triggered task-switching, also referred to as voluntary task-switching [15] or self-interruptions [13], where individuals determine the timing of task transitions. Various cues can trigger a switch, including the time elapsed since the last switch [16], completion of subgoals [17], engagement in a task that is no longer rewarding [17], and the perceived difficulty or priority of a task [14,16,18]. Furthermore, a mismatch between task difficulty and an individual’s capabilities may trigger task-switching. Tasks perceived as exceedingly easy can lead to increased switching to counteract boredom, while tasks regarded as highly challenging may also prompt more frequent switching to alleviate fatigue [13]. Empirical research indicates that multitasking, while associated with increased error rates and diminished performance, is a prevalent behavior among humans who often even prefer multitasking over the sequential completion of tasks [11,12,17,19].

While multitasking can get taxing for humans already, it is a huge challenge for RL agents. Generally, two promising approaches to facilitate goal-directed behavior for complex objectives are model-based RL and hierarchical RL [20]. *Model-based* RL enhances traditional RL by incorporating an internal representational model that predicts future states and rewards based on prior states and actions. This enhancement significantly augments an agent’s planning capabilities and allows for simulations, enabling strategic evaluations without direct interaction with the environment [4,21]. *Hierarchical* RL, on the other hand, organizes learning across various levels of abstraction, effectively simplifying complex tasks by breaking down the extensive state and action spaces into more manageable segments [4,22,23]. Hierarchical RL helps to address the challenge of serial multitasking by enabling the learning of sub-policies for distinct tasks and switching between them [24–26]. However, these sub-policies typically operate within the same environment while executing their specialized tasks [26,27]. Others adapted hierarchical RL to allocate a dedicated sub-agent for each task, with each operating in its own environment to support humans in multitasking challenges [28]. In this framework, while each sub-agent is responsible for a specific task, a higher-level agent determines the appropriate moments for transitioning between tasks. It has been argued that combining model-based RL and hierarchical RL unlocks an order-of-magnitude leap in agent capabilities. For example, it would allow the higher-level agent to leverage an internal model to predict the outcomes of lower-level agents [29,30]. Still, enabling serial multitasking behavior in scenarios with distinct tasks that are running continuously and in parallel remains a challenge for RL. In particular, it is not yet fully understood how to model the decision-making process required for switching between the continuous tasks in RL agents.

We argue that one can apply insights gained from understanding the mechanisms behind task-switching in humans to enhance autonomous agents’ decision-making processes in multitasking environments. Specifically, we posit that a *sense of control* (SoC) serves as a basic mechanism for integrating various factors influencing human task-switching. The SoC refers to the feeling of being in control over a situated action [31,32]. We distinguish between a *situational* SoC and a *general* SoC. The general SoC has been investigated in various human studies where participants evaluate their subjectively perceived feeling of control (e.g., using a Likert scale) at the end of a trial [33–36]. In contrast, the situational SoC is continuously evolving over time during situated action within a trial [31]. We assume the situational SoC to be very valuable for multitasking because it provides a crucial judgment of the current situation that may inform decisions about how and when to switch control.

Consider a traffic scenario. In a stop-and-go traffic scenario, the driver often faces the temptation to engage with their mobile phone. Here, the SoC serves as a critical metric for assessing the ability to alternate between the dual tasks of operating a vehicle and utilizing a smartphone. When an individual effectively manages both activities, the SoC for each task remains high, facilitating seamless transitions between driving and phone use. Conversely, if the traffic condition becomes problematic, for example, through frequent lane changes or the presence of numerous vehicles, the SoC related to driving diminishes. This reduction occurs because the driver must concentrate on monitoring the roadway and responding to dynamic conditions, thereby making it unlikely that they will divert attention to their phone. As traffic begins to ease, the SoC for driving correspondingly increases. Moreover, in instances where the vehicle stalls while starting, the driver experiences a significant drop in SoC, necessitating immediate focus on rectifying the starting issue before any other activities are considered. Once the situation has been addressed, the SoC for driving rises again, potentially prompting the driver to shift their attention to their mobile device. When the driver engages with their phone, their awareness of the driving environment diminishes. The inability to assess the current traffic conditions leads to a further decline in the SoC associated with driving. Ultimately, as the SoC for driving reaches a critically low threshold, the driver is compelled to return their focus to the roadway to ensure proper monitoring and safe navigation in the stop-and-go traffic condition. Re-engaging with the driving task effectively restores the SoC for that activity, enabling informed decision-making regarding subsequent actions.

This example suggests that an SoC may serve as a significant cue and indicator of the current circumstances, particularly in relation to task-switching decisions associated with multitasking. Additionally, research has highlighted the SoC’s critical role in action control, whereby multitasking can be viewed as a specific instance of complex action control [31,37,38]. In the following, the term SoC will be used to refer specifically to the situational SoC.

Modeling and applying an SoC in autonomous RL agents is an open research area. A promising approach for simulating an SoC is based on a *prediction error* assessing the discrepancy between what the agent predicts and perceives as the state of the environment [31,32]. Prediction errors are frequently used for action control and are well-established in other fields such as model predictive control and predictive coding [39,40]. However, we argue that a sole focus on prediction error is insufficient for describing the SoC. In environments characterized by dynamic complexity and uncertainty, situations that can be accurately predicted can nonetheless diminish the perceived SoC. For instance, scenarios that necessitate numerous interventions and corrections often contribute to a reduced SoC [36]. Therefore, we propose an additional component termed *need for control*. This component relates to the difference in rewards obtained while following a current policy (active policy) versus the rewards that would be obtained even in the absence of control over the task (inactive policy). A substantial discrepancy in rewards indicates a high need for control, suggesting the necessity for intervention. Thus, we propose a model of SoC that comprises both the comparison of intended and perceived outcomes (the prediction error component) and the interventions and actions needed (the need for control component). We posit that this integrated model more adequately accounts for various factors influencing human task-switching. For instance, factors that trigger a task-switch, such as the completion of subgoals, which results in a diminished need for control, or the difficulty of the task, which leads to increased prediction errors in more challenging tasks, are comprehensively represented.

The overall objective of the present work is to incorporate an SoC into the decision-making processes of RL agents to facilitate the learning of task-switching policies that increase the performance in multitasking environments. To that end, we introduce a hierarchical RL setup designed to address multitasking challenges and extend it with the above-described model of a situational SoC. The obtained results demonstrate that including a situational SoC in AI agents can lead to superior and even superhuman performance in such demanding control scenarios. Our detailed contributions are as follows:

- We describe a specific, implemented multitasking scenario that consists of multiple, concurrent instances of a *Collect Asteroids* task. In the Collect Asteroids task, an agent is required to navigate a spaceship through a spatial environment while collecting asteroids. Within our multitasking framework, two instances of the Collect Asteroids task are running concurrently, yet only one task can be perceived and controlled at any given time. The objective for the agent is to develop an effective task-switching strategy that maximizes the cumulative reward across both tasks. This custom multitasking arrangement effectively merges the necessity for task-switching inherent to serial multitasking with the attributes of concurrent multitasking.
- We present a hierarchical RL architecture designed to tackle serial multitasking scenarios. At a lower level, it consists of sub-agents dedicated to solving an individual Collect Asteroids task; at a higher level, a meta-agent directs the task-switching process and determines which sub-agent is permitted to actively engage in and observe its respective task, while the remaining task follows the inactive policy.
- We propose a mathematical formulation of a situational SoC, based on prediction error and need for control. An internal model utilized by the RL agents is leveraged to generate predictions concerning future states, which in turn facilitates computing both the prediction error and the need for control. The prediction error is derived by comparing the anticipated state as predicted by the internal model with the accomplished state when taking the action. The need for control employs the internal model to simulate the future using either the control policy or the inactive policy, followed by comparing the respective rewards obtained with each policy. The SoC model is employed by the meta-agent to enhance its switching behavior between the tasks.
- We report a study evaluating the effectiveness of the proposed approach. This involves a comparative analysis of the task-switching behavior and performance of our RL agents with and without an SoC, as well as with human participants acting in the same multitasking scenario.

## Materials and methods

To study how accounting for an SoC in AI agents can help to enhance their performance in multitasking scenarios, we propose an RL setup in which those agents can be trained to improve their decision-making on task-switching. This section outlines the multitasking scenario employed in our study, detailing both the specific subtasks and the overarching multitasking structure. Further, we present an agent architecture for this scenario, composed out of sub-agents responsible for individual subtasks and a meta-agent coordinating the multitasking, along with an internal forward model utilized for generating predictions. In addition, we describe in detail a proposed SoC model, including a *prediction error* and a *need for control* component. We will then describe the overall decision-making process model and provide details on the training and the implementation of the agents. Finally, we describe the methods employed in a study conducted in the same setting with human participants for comparison.

### Scenario

We employ a multitasking setup in which the primary objective is to find an effective task-switching strategy to optimize the cumulative reward across two subtasks. Each subtask is a *Collect Asteroids* task, which involves maneuvering a spaceship to collect the maximum number of asteroids appearing in an unpredictable way. The following sections provide a detailed description of the subtasks and the overarching multitasking scenario.

#### Subtasks - *Collect Asteroids.*

While much of the current multitasking research focuses on discrete actions in environments that remain static over time, our study introduces a setup that emphasizes continuous motor-control tasks [41–43]. This approach integrates serial multitasking with the dynamics of continuous motor control. In our multitasking setup, agents and participants can manage and perceive only one task at a time, necessitating serial task-switching; nevertheless, the not-attended task runs concurrently and evolves continuously in the background. A recent study by [28] has explored a variation of this novel setup, however, with a focus on assisting human task-switching behavior rather than on enabling AI agents to master this multitasking challenge themselves. The objective of such an agent is to optimize the overall scoring over both subtasks that are running concurrently, by strategically switching between the tasks in a serial manner.

**Fig 1:**
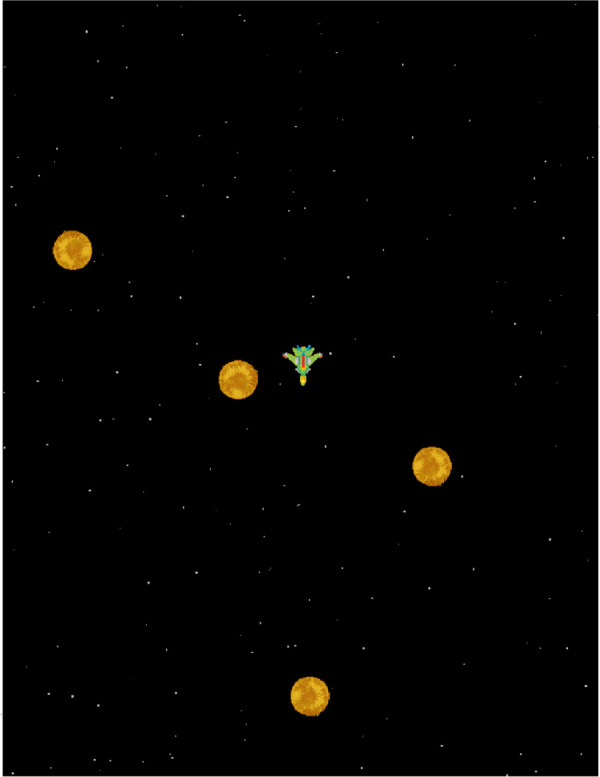
Spaceship flying through the Collect Asteroids world. The figure depicts a representative situation that may be encountered during gameplay in the *Collect Asteroids* game. The goal is to collect the asteroids (displayed in gold) with the spaceship.

The *Collect Asteroids* subtask involves maneuvering a spaceship through a defined environment. In the environment, the spaceship automatically moves downward, while the player/agent can change its horizontal position by steering to the right or left. Walls on both sides set boundaries of the environment. Between the walls, the space is populated with randomly distributed asteroids. The primary goal is to collect as many asteroids as possible. Fig 1 shows an exemplary situation during this task.

In the multitasking framework, two *Collect Asteroids* subtasks have to be carried out in parallel. Each subtask can be adjusted to one of two levels of *difficulty*: easy and hard. The difficulty influences the number of asteroids present in the environment; specifically, the easier configuration contains 14 asteroids, whereas in the hard condition, there are 30 asteroids. In addition to difficulty, we induce uncertainty by adding stochastic *input noise* to the execution of steering actions. In the absence of input noise, a left or right action results in the corresponding movement of the spaceship by one distance unit. However, when input noise is applied, an offset derived from a normal distribution with a mean μ=0 and a variance $\sigma^{2}=1.5$ is added to the resultant left or right move. Each iteration of both subtasks consists of 470 steps.

We opted to utilize a custom game as a problem scenario for several key reasons. First, it is essential that we can effectively manipulate a sense of control. Cognitive and psychological research has shown that a sense of control can be adjusted through variations in task difficulty and the introduction of prediction errors [32,36]. In the *Collect Asteroids* task, we can manipulate difficulty by altering the quantity of asteroids present in the environment. Additionally, we create prediction errors by incorporating input noise into the action input, as previously described. Second, the subtasks are constructed to facilitate training AI agents through limited state, observation, and action spaces, as well as deterministic actions when input noise is not applied. Third, due to a fixed number of asteroids for each difficulty level, performance assessment becomes straightforward. More specifically, it is quantified using a *success rate* (*SR*), which is calculated by dividing the number of collected asteroids ($\#\;{ast}_{collected}$) by the total number of asteroids (# ast): $SR=\frac{\#\;{ast}_{collected}}{\#\;ast}$.

#### Multitasking.

The multitasking scenario is created by having to execute two instances of the *Collect Asteroids* subtask concurrently, each of them with possibly different levels of difficulty or input noise. Consequently, we categorize the scenarios into three combinations of difficulty: easy + easy, easy + hard, and hard + hard. Within each of these combinations, we can further identify variations based on the presence of input noise, which might be absent in both tasks, present in only one task, or present in both tasks. Specifically, for the easy + hard combination, we can distinguish between input noise affecting the easy task versus the hard task. This yields a total of ten distinct multitasking configurations. Both tasks are running concurrently, but only one of them can be attended to and controlled at any point in time. The primary objective of the multitasking scenario is thus to develop an effective task-switching policy that optimizes overall reward.

### Agent model

**Fig 2:**
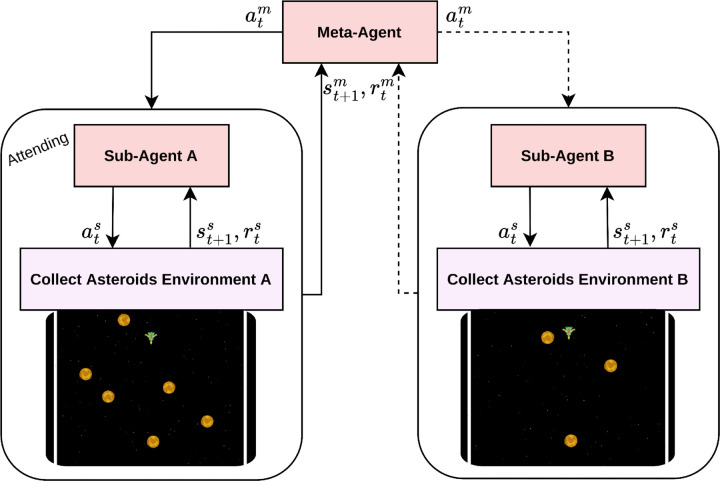
Hierarchical architecture with sub- and meta-agents. The figure displays the hierarchical setup of two sub-agents at the lower level and a meta-agent at the higher level. Here, the meta-agent selects sub-agent A, which is represented by solid arrows, while the connections to sub-agent B are indicated with dashed arrows. Note that actions, states, and rewards associated with the sub-agents are denoted with the superscript “s”, whereas those related to the meta-agent are specified with the superscript “m”.

To address the scenario outlined above, we need to lay out an architecture for an AI-based agent that can autonomously learn how to act in a multitasking setting. The goal is to facilitate successful switching between the two continuous subtasks while maintaining optimal performance within each subtask. Notably, this entails two distinct learning objectives: firstly, to establish an optimal policy for each subtask aimed at collecting a maximum of asteroids; and secondly, to devise an effective policy for switching between the subtasks. Given the inherent limitation of addressing only one task at a time, the switching strategy must be designed to optimize the total number of asteroids collected. In light of these differing objectives, we develop two kinds of agents. The first category consists of sub-agents dedicated to the individual subtasks, i.e., optimized for collecting asteroids. Since our multitasking scenario encompasses two subtasks, we implement two sub-agents, one for each task, characterized by similar design parameters. The second category is a meta-agent tasked with orchestrating the switching between the two subtasks. Together, these two types of agents are organized within a hierarchical RL architecture, where the meta-agent determines which sub-agent is currently authorized to act. The hierarchical structure is shown in Fig 2.

#### Sub-agent.

The sub-agents are designed as RL agents with the objective of collecting asteroids. Such RL problems characterized by fully observable states can be formally described as a Markov Decision Process (MDP) defined by four key components: (*S*, *A*, *P*, *R*). The set *S* encompasses all potential *states s*. In this specific subtask, a state s∈S is represented as an array that encompasses the positional coordinates of the spaceship and the asteroids, thereby reflecting the current world state of the game. The *action* space *A* consists of all feasible actions *a*. The sub-agent has three possible actions: a∈A={0,1,2}, where action 0 corresponds to moving one step to the left on the grid, action 1 signifies no steering at all, and action 2 represents moving one step to the right. At each time step, the agent also updates its position by moving one step down in the environment. The *transition model* $P(s,a,s^{\prime })$ → [0, 1] characterizes the likelihood of transitioning from state *s* to state $s^{\prime }$ as a result of action *a*. In the absence of input noise, this transition model remains deterministic; however, the transition model becomes non-deterministic and hence probabilistic in the presence of input noise. The *reward* function *R*(*s*, *a*) → ℝ defines the reward *r* obtained when the agent performs action *a* in state *s*. Our implementation of *R*(*s*, *a*) is composed of two distinct components. First, the positional state *s* is converted into a simplified image representation that illustrates the spaceship, the asteroids, and the walls. Subsequently, a Gaussian filter is applied to this simplified representation. The blurring effect of this Gaussian filter enhances the reward for regions surrounding the asteroids, thereby inducing the agent to pursue these targets. The reward is calculated based on the value of each grid position where the spaceship is depicted in the image. Second, a distance-based reward is added to minimize the horizontal distance between the spaceship and the incoming asteroids, promoting early alignment with these objectives. Such distance-based reward design is particularly beneficial in multitasking environments where actions (here, asteroid collection) must be prepared as early as possible due to the possibility of switching to another subtask and losing control of the current one.

#### Meta-agent.

The meta-agent’s primary function is to determine which sub-agent can take action at any given timestep. While the sub-agents possess complete visibility of their respective states, the meta-agent is limited to observing only the active task it has selected. Consequently, this RL agent is characterized by a partially observable Markov decision process (POMDP) [44]. A POMDP encompasses six components: *S*, *A*, *P*, *R*, Ω, and *O*. In this framework, *S*, *A*, *P*, and *R* are defined in a manner analogous to a conventional MDP as described above. This means, the *states* s∈S reflect the real-world state of the game, being the situation of both subtasks. The *states* s∈S represent the actual conditions of the game, reflecting the situation of both subtasks. Regarding *actions*, we define a∈A={0,1}, indicating the currently active task. Specifically, if *a* = 0, the sub-agent responsible for the first task is permitted to execute its operations; conversely, if *a* = 1, the sub-agent for the second task is granted control. The *transition model* $P(s,a,s^{\prime })$ → [0, 1] quantifies the probability of progressing from state *s* to state $s^{\prime }$ as a consequence of executing action *a*. In terms of *reward* structure, we experimented with multiple reward functions *R*(*s*, *a*) → ℝ applicable to the meta-agent. The first is the *active reward function*, wherein the reward for the meta-agent is solely derived from the subtask reward associated with the currently active sub-agent. The second, termed the *SoC reward function*, is shown in Eq 1. Under this function, the meta-agent receives a positive reward of 1 for selecting the sub-agent corresponding to the action with the lower SoC, a negative reward of –1 for selecting the sub-agent with the higher SoC, and a neutral reward of 0 otherwise. This approach rewards turning to the subtask that the agent “senses” is less well under control and hence needs more attention.

$$r_{t}^{meta}=\left\{ \begin{array}{cc} -1 & \;\text{if }{SoC}_{0}>{SoC}_{1}\text{and }a=0\text{ or if }{SoC}_{0}<{SoC}_{1}\text{and }a=1\\ 0 & \;\text{if }{SoC}_{0}={SoC}_{1}\\ 1 & \;\text{if }{SoC}_{0}>{SoC}_{1}\text{and }a=1\text{ or if }{SoC}_{0}<{SoC}_{1}\text{and }a=0 \end{array}\right.$$ (1)

The set Ω comprises all *observations o* available to the agent. This distinction implies that a POMDP differentiates between the actual true state *s* and any kind of observables represented by *o*. Given that the true state *s* associated with each task remains unknown, a belief state probability *b*(*s*) → [0, 1] is employed to represent the agent’s degree of belief in the true state. It is thus given as a probability distribution over all states s∈S, satisfying the condition $\sum_{s\in S}b(s)=1$ and representing how likely the world is in the corresponding state according to the agent’s subjective state of knowledge. The objective of the meta-agent is to determine the optimal moments for switching between two tasks. Because we model this problem as a POMDP, the observation o∈Ω is defined as a concatenation of the true state *s* of the currently active task and a belief state *b* about the inactive task. Various observation formats were evaluated for the meta-agent. In one approach, the observation encompasses the true state of the active task alongside the belief state of the inactive task, which is represented by positions of the spaceship and asteroids and therefore called *world state observation*. Alternatively, the observations may encompass the agent’s SoC with regard to the active or inactive tasks (see below for how the SoC is calculated) and is called *SoC observation*. Due to comparison with human players engaging in the same game, we also established that the meta-agent is permitted to switch tasks not more often than every five frames, in line with previous similar studies indicating that humans can execute rapid switches within a time frame of half a second [45]. This implies that following each switch, the sub-agent is required to perform at least five actions before the meta-agent is authorized to switch to the other subtask. The correspondence between states and observables is defined by $O(s^{\prime },a,o)$ → [0, 1] as the *conditional probability* (likelihood) to receive observation *o* after executing action *a* in state $s^{\prime }$.

To solve the (PO)MDP, the agent aims to learn the optimal policy $\pi (a|s{)}^{*}$, which seeks to maximize the expected reward across all states *s*. The policy π(a|s) represents the probability of selecting action *a* given state *s*, adhering to the condition that $\sum_{a\in A}\pi (a|s)=1$.

#### Forward model.

In addition to the sub- and meta-agents, we utilize an internal forward model to predict the state and reward of the environment for specific subtasks. This forward model takes as input the current environmental state, represented as an array indicating the positions of the spaceship and asteroids, along with an action corresponding to the intended movement. The output generated by the model is a predicted resulting state, which is also represented in array format detailing the updated positions of all relevant entities in the environment as well as the reward. At present, we employ a straightforward mathematical approach to calculate the next positions. Specifically, all y-positions are decreased by 1, consistent with the progression in time, while the x-positions are adjusted based on the specified action. An action value of 0, which corresponds to a movement to the left, results in a decrement of the x-position by 1. Conversely, an action value of 2, indicating movement to the right, results in an increment of the x-position by 1. An action value of 1 entails no change to the x-position. Looking ahead, it is anticipated that the forward model will be implemented by a neural network trained to effectively predict the next state based on a broader set of inputs and learned patterns. Note that the current forward model operates without any information regarding input noise and, as a result, lacks the ability to account for the effects introduced by such noise.

### Modeling sense of control

The key hypothesis of the present work is that action control in multitasking environments entails decision-making related to task-switching, and that a proper mathematical model of a situational SoC may facilitate the learning of corresponding strategies in AI agents. In psychology, the SoC is a construct representing an individual’s feeling of being in control of a specific action within a situation. This concept is seen as a component of the broader *sense of agency* (SoA), which encompasses the awareness of being the initiator of an action. It is suggested that the SoC develops through human interactions with the environment [31].

A common approach to model SoC and SoA is based on the computation of the discrepancy between a predicted state generated by an internal model and the actually perceived state, a concept known as the *comparator model* theory [31,46,47]. While this theory has received support from various empirical studies examining the SoA (for comprehensive reviews, see [32,48,49]), it has also faced criticism for its narrow focus on sensorimotor mechanisms. This critique is particularly relevant given that the SoA can be shaped by diverse alternative cues [32,49]. In light of this limitation, the *multifactorial weighing model* has been introduced, proposing that distinct SoA cues are assigned weights based on their reliability within a specific context, thus influencing the formation of the SoA [32]. Research involving human participants has demonstrated that humans form an SoC for each subtask involved in a multitasking scenario and that these subtask SoCs are affected by factors such as input noise, which generates prediction errors, as well as task difficulty, which imposes cognitive load and raises a need for controlling the situation [36,45]. Other influences, such as feedback following task performance, have been found to impact reported SoC in some studies [34], yet have not been substantiated in others [45], such that the specific array of agency cues remains a subject of ongoing research [50].

To the best of our knowledge, there exists no established mathematical model to compute the SoA or SoC. Nevertheless, similar concepts, particularly the computation of prediction errors, have been developed and applied across multiple fields. An illustrative case is predictive coding, which posits that the human brain creates an internal model of its external environment to predict sensory inputs and uses them to plan and control actions. In this paradigm, alignment between actual sensory data and predicted inputs enables the filtering of redundant information, whereas discrepancies reveal novel and unexpected environmental states. However, the mechanisms for calculating prediction errors in real-world scenarios and for updating predictive models to minimize such errors continue to be subjects of active research [40,51]. Additionally, in control theory, model predictive control employs internal models to foresee future states. The aim is to minimize the prediction error between the model’s projections and the actual outcomes of the control process, thereby achieving minimal costs as defined by a cost function [39,52].

Research indicates that the SoC in humans can be influenced by prediction errors and the complexity of tasks [32,53,54]. Furthermore, various disciplines have demonstrated the relevance of prediction errors in the development of intelligent agents. Building on these insights, we propose a mathematical model for computing an SoC for the active subtask based on two components: (1) the current prediction error in controlling the subtask and (2) the need for controlling the subtask, which is taken to be moderated by task difficulty.

#### Preliminaries.

Before defining a model for computing an SoC, we lay down the mathematical notation for essential concepts. Let *a*_*t*_ denote the action executed at time step *t*, and let *s*_*t*_ represent the corresponding state at that time step. The environment, denoted as *e*, receives *a*_*t*_ as input while in state *s*_*t*_ at time *t*. Upon executing the action *a*_*t*_, a new state *s*_*t* + 1_ is generated. Concurrently, a reward *r*_*t*_ is computed to assess the effectiveness of taking action *a*_*t*_ in state *s*_*t*_ (according to Eq 2).

$$s_{t},a_{t}\overset{e\;}{\to }s_{t+1},r_{t}$$ (2)

Furthermore, an internal forward model *m* similarly utilizes *a*_*t*_ and *s*_*t*_ as inputs. The output of this forward model is the predicted subsequent state, denoted as ${\hat{s}}_{t+1}$ (see Eq 3).

$$s_{t},a_{t}\overset{m\;}{\to }{\hat{s}}_{t+1}$$ (3)

#### Prediction error.

We introduce input noise in our experimental setup to create prediction errors and hence likely variations in the SoC. Input noise refers to the perturbations in the input provided by the agent. Specifically, when the agent selects an action *a*_*t*_ to move left or right, a noise value is added to the intended step. This input noise is sampled from a normal distribution characterized by a mean μ=0 and a variance $\sigma^{2}=1.5$. Notably, the forward model utilized in our study is unaware of the existence of input noise, which results in a discrepancy when comparing the predicted state ${\hat{s}}_{t+1}$ with the actual state of the environment *s*_*t* + 1_; this disparity leads to a prediction error.

The prediction error ${PE}_{t}$ is defined as the difference between a model’s prediction and the actual outcome. Accordingly, it is computed as the difference between the actual next state *s*_*t*_ and the predicted next state ${\hat{s}}_{t}$, as outlined in Eq 4.

$${PE}_{t}=\Delta (s_{t},{\hat{s}}_{t})$$ (4)

In the given scenario, the state *s*_*t*_ at time step *t* is defined by the positions of both the spaceship and the asteroids present. Notably, the input noise is exclusively applied to the spaceship and solely affects the *x*-coordinate step size. Consequently, we focus on the actual *x*_*t*_ coordinate of the spaceship alongside its predicted coordinate, ${\hat{x}}_{t}$, in order to compute the prediction error ${PE}_{t}$ as the Euclidean distance *d*_*t*_ between the actual position *x*_*t*_ and the predicted position ${\hat{x}}_{t}$.

To restrict the prediction error ${PE}_{t}$ to the interval [0, 1]: ${PE}_{t}\in [0,1]$, it is essential to normalize the distance *d*_*t*_. A commonly used method for normalization is min-max normalization [55]. This technique involves subtracting the minimum value from the data point being normalized and then dividing the result by the range of the dataset. A min-max normalization was deemed unsuitable due to noise in the input, sampled from a normal distribution with a mean of μ=0 and a variance of $\sigma^{2}=1.5$. Consequently, over 95% of the input noise values are contained within the range of [–3, 3]. However, the maximum theoretical distance can be much higher, but is rarely realized in practice. If a min-max normalization would be applied, the predominant distances would be on the order of 3, resulting in consistently small prediction error values. Therefore, we have opted to utilize a hyperbolic tangent function tanh to normalize the prediction error (see Eq 5). Following Eq 5, all distances *d*_*t*_> = 4 have a prediction error approximately equal to 1, while distances *d*_*t*_<4 range between 0 and 1.

$${PE}_{t}(x_{t},{\hat{x}}_{t})=tanh\left(0.5*(|x_{t}-{\hat{x}}_{t}|)\right)$$ (5)

It is important to recognize that a prediction error can stem from two primary sources. The first is known as *epistemic uncertainty*, which occurs when the forward model is inadequately trained. In this scenario, the predicted state does not accurately reflect the true state, resulting in a substantial deviation during the comparison. This type of uncertainty can be mitigated through the implementation of a well-trained forward model. The second source is referred to as *aleatoric uncertainty*, which arises when the actual state is novel and has not yet been encountered by the forward model. Unlike epistemic uncertainty, aleatoric uncertainty is inherently irreducible [56]. For the purpose of this analysis, our focus will be on *aleatoric uncertainty*, because we can rely on the exact mathematical calculation of the next state as a forward model.

#### Need for control.

Recent research indicates that cognitive load, influenced by varying levels of difficulty, impacts the SoC in individuals [36,45]. Accordingly, we implemented two levels of difficulty, designated as *easy* and *hard*, for each subtask. These levels impose different degrees of effort required to accomplish the task. Specifically, a harder task involves collecting a higher number of asteroids, necessitating more actions to achieve the goal of maximizing asteroid collection. We define the amount of actions required in this context as the *need for control*. When the agent has to engage in numerous actions to collect an asteroid, its need for control is heightened; conversely, this need diminishes when no asteroids are displayed.

To quantify the need for control, we analyze the rewards generated by two distinct trajectories in the active sub-environment: one following the optimal policy, i.e., given by an efficiently trained RL agent that persistently acts on the subtask, and the other resulting from performing the default action only, thus corresponding to an agent that does not actively attend to and act on the subtasks. Furthermore, we assign weights to the rewards at each step, prioritizing near-future rewards over those in the distant future. The principle underlying this approach is that when the reward sequences from these two trajectories diverge significantly, it suggests a heightened need for control.

The trajectories, each of length *N*, comprise sequences of states paired with their corresponding rewards: ($\left(s_{t},r_{t-1}),({\hat{s}}_{t+1},{\hat{r}}_{t}),...,({\hat{s}}_{N-1},{\hat{r}}_{N-2}\right)$). To construct the trajectories, we utilize a forward model, denoted as *m*, to compute the subsequent states and rewards. Let *s*_*t*_ represent the current state and *a*_*t*_ the action taken. The next predicted state ${\hat{s}}_{t+1}$ and reward ${\hat{r}}_{t}$, is determined by inputting *s*_*t*_ and *a*_*t*_ into the forward model *m*. For subsequent predicted states, ${\hat{s}}_{t+i+1}$ and rewards ${\hat{r}}_{t+i}$, the last predicted state, ${\hat{s}}_{t+i}$, along with the action *a*_*t* + *i*_, is fed into *m* The trajectory pairs are collected for steps where *i* < *N* – 1.(refer to Eq 6).

$$3s_{t},a_{t}\overset{m\;}{\to }{\hat{s}}_{t+1},{\hat{r}}_{t}\;\;{\hat{s}}_{t+i},{\hat{a}}_{t+i}\overset{m\;}{\to }{\hat{s}}_{t+i+1},{\hat{r}}_{t+i}\;\forall \;i\in [1,N-2],i\in \mathbb{N}$$ (6)

Both the default and optimal trajectories compute their respective sequences of states and rewards as previously described, albeit through distinct actions. The default trajectory relies solely on the default action, whereas the optimal trajectory incorporates actions determined by a perfectly trained agent.

To assess the need for control, the rewards from the reward sequences of both the default and optimal trajectories are aggregated and adjusted using a weighting factor γ=0.9 (see Eq 7). This results in the reward sum for the default trajectory, ${rs}_{t}^{d}$, and the reward sum for the optimal trajectory, ${rs}_{t}^{o}$.

$${rs}_{t}=r_{t-1}+\sum_{k=1}^{N-1}\gamma^{k}{\hat{r}}_{t+k-1}$$ (7)

Finally, the need for control ${NfC}_{t}$ at time step *t* is calculated by taking the difference between these two sums: specifically, by subtracting the rewards gathered through the optimal policy from those obtained via the default policy, and subsequently dividing the result by 100. The reward associated with the optimal trajectory ${rs}_{t}^{o}$ is always equal to or exceeds that of the default trajectory ${rs}_{t}^{d}$ (${rs}_{t}^{o}\geq {rs}_{t}^{d}$), which eliminates the necessity for establishing lower bounds. Finally, the value of ${NfC}_{t}$ is constrained between 0 and 1, ensuring that ${NfC}_{t}\in [0,1]$ (refer to Eq 8).

$${NfC}_{t}=\min\left(\frac{{rs}_{t}^{o}-{rs}_{t}^{d}}{100},1\right)$$ (8)

When the cumulative rewards of both the default and optimal trajectories are similar, the resultant difference is minimal, yielding a need for control ${NfC}_{t}$ close to 0. Conversely, when there is a significant disparity between the reward sums of the two trajectories, the difference is substantial, leading to a high need for control ${NfC}_{t}$.

#### SoC.

We leverage the prediction error and the need for control to formulate an SoC. This approach aligns with the multifactorial weighting model, which posits that various cues influence the SoC [32]. Drawing upon a range of studies, we focus specifically on the cues of prediction error and need for control to provide a mathematical description of the SoC [36,45].

The SoC at timestep *t* (${SoC}_{t}$) is computed using the prediction error (${PE}_{t}$) and the need for control (${NfC}_{t}$). In examining prediction error, a high value signifies a significant discrepancy between the anticipated and actual outcomes, resulting in a diminished feeling of control. Conversely, a low prediction error indicates a close alignment between expectation and reality, fostering a heightened feeling of control. Regarding the need for control, a high need correlates with a reduced feeling of control, whereas a low need, suggesting minimal action required, contributes to a stronger sense of control. Consequently, the ${SoC}_{t}$ is derived by subtracting the average of the ${PE}_{t}$ and the ${NfC}_{t}$ from 1. Both ${PE}_{t}$ and ${NfC}_{t}$ are constrained within the interval [0, 1], ensuring that the SoC also falls within this range (see Eq 9).

$${SoC}_{t}=1-\frac{{PE}_{t}+{NfC}_{t}}{2}$$ (9)

In the context of our multitasking setup, control is limited to only one active task at a time, which restricts the calculation of ${PE}_{t}$ and ${NfC}_{t}$ to that specific task while leaving the agent without precise knowledge of the status and outcome of the other task. Given findings indicating that humans assess an SoC for each task during multitasking, it is necessary to estimate the SoC (${\hat{SoC}}_{t}$) for the unattended task [45]. This estimation incorporates the prior SoC ${SoC}_{t-1}\in [0,1]$ at the preceding time step, decrementing it by a variable ϕ=0.1. A maximum operator is applied to guarantee that ${\hat{SoC}}_{t}$ does not fall below zero. The equation governing the SoC of the inactive task is shown in Eq 10.

$${\hat{SoC}}_{t}=max(0,{SoC}_{t-1}-\phi )$$ (10)

This conceptual model for the SoC equips the agent with the capability to navigate complex multitasking scenarios and enhances decision-making regarding task-switching.

### Decision-making process

**Fig 3:**
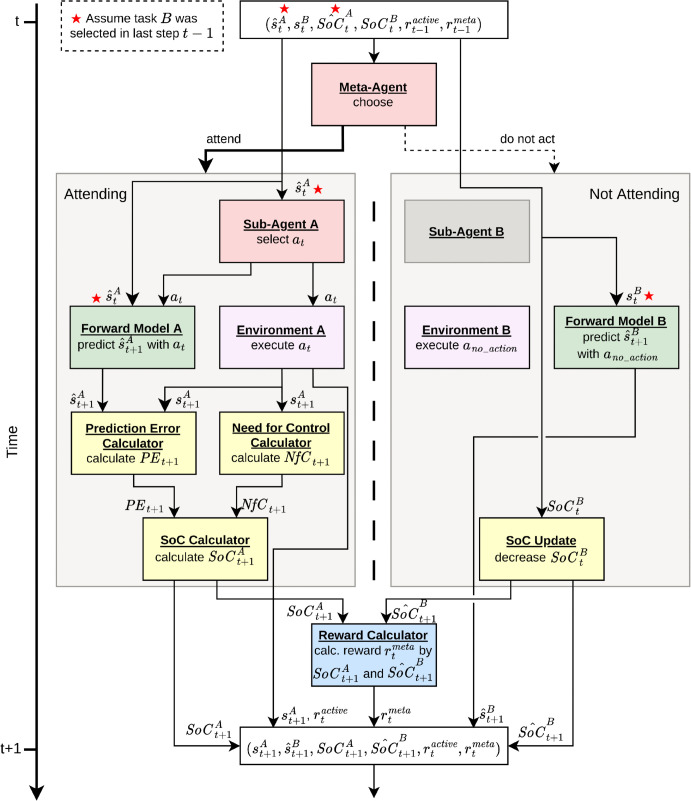
Process model. The process model at timestep *t* is defined as follows: Given the most recent observation, the meta-agent selects a sub-agent to execute control during timestep *t*. While the designated sub-agent performs actions within the environment and concurrently has its SoC computed, the alternative sub-agent stays inactive and consequently experiences a reduction in its SoC.

We now turn to our approach to integrate the sub-agents, the meta-agent, and the SoC into a larger AI agent that shall autonomously learn how to act efficiently and successfully in the multitasking scenario. To that end, we combine the various components into the above-mentioned hierarchical RL architecture that implements the decision-making process as shown in Fig 3. We structure this process into three RL agents arranged at two layers. At the lower layer, two sub-agents are dedicated to one *Collect Asteroids* subtask each. These RL agents (denoted RL Agent A and B in Fig 3) are designed to learn independently, without interfering with one another or the multitasking framework. Prior to their deployment in the multitasking context, these sub-agents are trained to navigate the spaceship through the environment while maximizing the collection of asteroids.

In the overall decision-making process, at each time step *t*, the meta-agent evaluates the current state, which may include either the true positions or the SoC in the active (attended) task, in conjunction with the predicted positions or SoC in the inactive task. Additionally, it assesses the perceived reward, which corresponds to either the active sub-agent’s reward or the defined SoC reward function. Based on this information, the meta-agent determines which sub-agent will take action in the forthcoming step. For illustration, if sub-agent A is selected, the sub-agent will choose five actions *a*_*t*_, which are executed within its designated environment A. Concurrently, the internal forward model processes the last state, which could be either a predicted or actual state, comprising the positions concerning subtask A and the selected actions *a*_*t*_ to forecast the subsequent state ${\hat{s}}_{t+1}$. The predicted next state ${\hat{s}}_{t+1}$ and the actual next state of the environment *s*_*t* + 1_ are then employed to compute the prediction error, as described in the previous section. Furthermore, the need for control is assessed starting from the next state *s*_*t* + 1_ obtained from the environment. These two components are utilized to calculate the sense of control ${SoC}_{t+1}^{A}$ for the currently active task A. Simultaneously, for subtask B, which was not selected, sub-agent B remains inactive, leading to five executions of a default action in the concurrently progressing environment B. Moreover, the internal forward model for task B forecasts the next state ${\hat{s}}_{t+1}^{B}$. The SoC for task B, ${SoC}_{t+1}^{B}$, is calculated by reducing the SoC determined at the last time-step ${SoC}_{t}^{B}$ as outlined above. The reward $r_{t}^{meta}$ is computed depending on the selected reward function. Upon receiving the SoCs or states from the sub-agents, along with the reward determined by the selected reward function, the meta-agent’s policy identifies which sub-agent will be permitted to act next. This iterative process continues until the episodes of the sub-agents are concluded.

### Training and implementation details

All three agents, the two sub-agents as well as the meta-agent, are trained using Reinforcement Learning (RL). Our RL setup is developed using Python [57]. Both the sub-environments and meta-environment leverage the gymnasium library, which is a successor to OpenAI Gym [58,59]. For the visualization of the environments, we utilized the PyGame library (https://github.com/pygame/pygame). The training of agents is conducted using the stable-baselines-3 RL algorithm library [60] included in the Scilab RL framework (https://scilab-rl.github.io/Scilab-RL). Given that we are operating within a discrete action and state space, the Proximal Policy Optimization (PPO) algorithm is employed for training [61]. Throughout the training phase of the sub- and meta-agents, we performed hyperparameter optimization, specifically focusing on the learning rate *α*, the discount factor *γ*, the Generalized Advantage Estimation (GAE) parameter *λ*, and the entropy coefficient. From each round of hyperparameter tuning, we selected the agent that exhibited the highest average reward during the training period for subsequent evaluation. The sub-agents undergo training for a total of 10,000,000 steps to ensure long enough training for properly learning a policy for the problem. The meta-agent needs much longer for each time step because of the different calculations needed for each subtask. Therefore, the meta-agent utilizing the active reward function is trained for 1,300,000 steps. The meta-agent with the SoC reward function is trained only for 10,000 steps because the learning curve already converges at this point. Note that the training of the sub-agents is completed prior to using them by the meta-agent during multitasking. All training processes are executed on a compute cluster equipped with NVIDIA A40 GPUs. The code for training and evaluating the agents can be found on Github: https://github.com/Annika10/Scilab-RL/tree/v1.0.

Statistical analysis is conducted using the Python libraries scikit-posthocs and SciPy [62,63]. The results are visualized with Matplotlib [64].

### Human study

To further validate the RL agents incorporating the SoC, we gathered data with participants acting in the same multi-tasking scenario, thereby facilitating a comparative analysis between our RL method and human performance. We draw on existing literature to assert that the gamification of laboratory studies enhances the realism of participant behavior, which supports our objective of gathering authentic real-world data across various scenarios [65]. The human study was designed as a within-subject study and received approval from the ethics committee of Bielefeld University. Prior to participation, all subjects provided written informed consent. The study was conducted from May 6, 2025, to June 6, 2025. A total of 23 participants were tested (consisting of 14 males, 8 females, and one person who preferred not to disclose their gender), with a mean age of 25.17 ± 5.79. All participants were students or university staff. For our analysis, we measured the success rate (dependent variable) and employed a one-way ANOVA for each block, which indicated medium to large effect sizes across all difficulty levels and input noise configurations (independent variables). A post-hoc power analysis was performed using G*Power, with a standard alpha error probability set at 0.05 and a participant sample size of 23. The results of the power analysis revealed a power range between 0.48 and 0.90, dependent on the respective effect sizes. The data collected was anonymized, ensuring that the identification of individual participants remains unattainable. The data and the analysis script are available at the Open Science Framework (OSF) website: https://osf.io/x5vtz.

The two instances of the *Collect Asteroids* games were presented on two Dell P2414H monitors, each with a resolution of 1920 × 1080 pixels. The games operated at a frame rate of ten frames per second, corresponding to ten discrete environmental actions occurring each second. Participants navigated the spaceships by pressing the [Y] key to move left and the [M] key to maneuver right, intentionally omitting the arrow keys to mitigate potential biases associated with left- and right-handedness. Transitioning between tasks required participants to press the [SPACE] key. Prior to the actual gameplay, participants were instructed to practice with the setup to eliminate any learning effects that might influence the study’s outcomes. A block is characterized by the specific difficulties of both tasks and the configured input noise, which results in ten blocks encompassing two trials. Each trial consisted of 470 steps/frames, resulting in a total trial duration of 47 seconds. The order of the blocks and of the trials within each block was randomized. After each trial, the participants proceed in a self-paced manner by pressing [ENTER] to start the next trial.

While our agents employed an intrinsic reward system, we opted for a simpler reward function for human participants in the *Collect Asteroids* task. This decision was based on the rapid pace of human gameplay, averaging ten moves per second, making it impractical for players to recognize and respond to complex rewards at each step. We thus implemented a sparse reward system, giving a negative reward for failing to collect an asteroid and a positive reward for successful collection. The magnitude of the reward is contingent upon the number of asteroids present in the game, which varies according to the selected difficulty level. Specifically, participants can achieve a maximum reward of 100 for collecting all asteroids, while a total penalty of -100 is assigned for missing all asteroids. The reward function for this subtask is formally defined in Eq 11, where *N* represents the total number of asteroids available in the game environment.

$$r_{t}=\left\{ \begin{array}{cc} m & \text{if collecting an asteroid in time step t}\}\\ -m & \text{if missing an asteroid in time step t}\}\\ 0 & \text{otherwise} \end{array}\right.\;\text{where}\;m=\frac{100}{N}$$ (11)

## Results

To investigate whether the integration of a situational SoC enhances performance in serial multitasking, we implemented, trained, and evaluated different variants of meta-agents controlling the same sub-agents. We start reporting the results by first assessing how the sub-agents independently solve their *Collect Asteroids* task. We then explore the performance of different meta-agents operating with different observational parameters and reward functions, with or without utilizing an SoC. After identifying the optimal configurations, we evaluate the corresponding hierarchical RL agent across varying levels of task difficulty and uncertainty. Finally, we conduct a detailed analysis of emerging task-switching strategies, for which each meta-agent configuration is evaluated using the same 100 episodes, with performance metrics quantifying the success rate (SR) as described above.

### Task performance of sub-agents

We conducted an evaluation of four configurations of sub-agents, each differing in difficulty levels and the presence of input noise. Specifically, we trained sub-agents on managing tasks categorized as easy and hard, both with and without input noise. For each combination, we performed a hyperparameter tuning and selected the sub-agent producing the best mean reward. The results, including the absolute number of asteroids collected and the corresponding success rates, are summarized in Table 1.

**Table 1:** Performance of sub-agents.

Configuration		Number of collected asteroids	
Difficulty	Input noise	Absolute	SR
easy (14 asteroids)	no	12.90 (± 0.89)	0.92 (± 0.06)
	yes	12.65 (± 1.02)	0.90 (± 0.07)
hard (30 asteroids)	no	24.05 (± 2.26)	0.80 (± 0.16)
	yes	22.93 (± 2.34)	0.76 (± 0.17)

Mean absolute numbers of collected asteroids and mean success rate (SR) per episode for each subtask configuration possible.

As shown in Table 1, the sub-agents exhibit effective performance across all tasks, with success rates ranging from 0.92 for the easier tasks without input noise to 0.76 for the more challenging tasks that include input noise. Notably, the introduction of input noise led to a decrease in success rates by two to four percentage points, while transitioning from easy to hard difficulty resulted in a drop of twelve to fourteen percentage points in the success rate. Due to the randomized placement of asteroids, we cannot guarantee that the optimal performance, i.e., collection of all asteroids, is feasible. Nevertheless, a success rate exceeding 90% for the easiest configuration indicates that the training methodology employed for the sub-agents was effective. We hence employ these trained sub-agents within the multitasking framework, in which two of them operate concurrently with task-switching by a meta-agent.

### Task performance of meta-agent

In order to assess a meta-agent, we need to evaluate its strategy for determining at each time step which sub-agent is best suited to maximize the total number of asteroids collected, when the two tasks are running at the same time. As outlined in the Methods section, we explore various configurations of the meta-agent. Specifically, we let it utilize two types of observations: the *world state observation*, which encompasses the current positions of the active sub-agent along with the predicted positions of the inactive sub-agent, and the *SoC observation*, which includes the calculated SoC of the active sub-agent alongside the predicted SoC of the inactive sub-agent. Furthermore, we implement two distinct reward functions: the *active reward function*, derived from the reward of the active sub-agent, and the *SoC reward function*, which evaluates the SoCs associated with both tasks. This yields four potential configurations for the meta-agent: the *no-SoC* agent, the *SoC-as-observation* agent, the *SoC-as-reward* agent, and the *SoC* agent. The *no-SoC* agent utilizes observations of the world state alongside an active reward function. In contrast, the *SoC-as-observation* agent relies on SoCs as observations. The *SoC-as-reward* agent employs the world state as observational data, with rewards determined by the SoC reward function. Consequently, the *SoC* agent gets SoC observations, with rewards assigned based on the SoC reward function. Due to the variability of the reward functions, a direct comparison of rewards is not feasible; instead, we assess these agents based on their SR. The evaluation is conducted under conditions where both subtasks are in an easy condition, and no input noise is present. The results are summarized in Table 2.

**Table 2:** Performance of different meta-agent configurations.

Agent	SR
No-SoC	0.70 (± 0.15)
SoC-as-observation	0.52 (± 0.12)
SoC-as-reward	0.68 (± 0.13)
SoC	**0.82 (± 0.11)**

Success rates (SR) for the different meta-agents: the *no-SoC* agent with the world-state as observation and the active reward function, the *SoC-as-observation* agent with an SoC as observation and the active reward function, the *SoC-as-reward* agent with an SoC as reward and the world state observation, and the *SoC* agent with an SoC as observation and reward.

Analyses reveal that incorporating the SoC solely as either an observation or a reward does not enhance decision-making in task-switching. This can be due to the difficulty in differentiating for the agent how the reward interacts with the observation. However, the results indicate that the meta-agent’s performance in task-switching is optimized when the SoC is utilized both as an observation and as a reward. Consequently, we aimed to further investigate by comparing two distinct agents: the *no-SoC* agent, which does not incorporate the SoC, and the *SoC* agent, which uses the SoC as an observation as well as defining the reward function.

We evaluated both these agents against two baseline conditions. The first baseline *switch-every-frame* is an agent that switches tasks at every possible interval, meaning it changes every five frames, since the meta-agent is restricted to switching only after the selected sub-agent has completed five actions related to its assigned task. The second baseline consists of *human* players engaging with the same game to provide for a comparative analysis of the agents’ performance.

The scenario under consideration drafts two distinct types of difficulty, leading to the formation of three primary blocks. The first block comprises both tasks configured at an easy level; the second block features one task at an easy level and the other at a hard level; and the third block consists of both tasks set to a hard configuration. Within each of these blocks, we introduce three different input noise conditions: no input noise, input noise in one task only, and input noise present in both tasks. Note that in the block where one task is easy, and the other is hard, we make a further distinction based on whether the input noise is applied to the easy task or the hard task. Consequently, this configuration leads to a total of ten unique conditions. The conditions are described using the format: difficulty of task one, difficulty of task two, and presence of input noise, separated by underscores: easy_easy_no, easy_easy_yes-in-one-task, easy_easy_yes, easy_hard_no, easy_hard_yes-in-easy-task, easy_hard_yes-in-hard-task, easy_hard_yes, hard_hard_no, hard_hard_yes-in-one-task, and hard_hard_yes.

To further evaluate performance differences among various conditions, we conducted a one-way ANOVA for each block to assess whether significant discrepancies exist between the *no-SoC* agent, the *SoC* agent, the baseline condition *switch-every-frame*, and *human* participants. All distributions displayed normality except for one. We tested homoscedasticity of variance across the data sets using Bartlett’s test, resulting in five violations [66]. ANOVAs are sufficiently robust to tolerate a limited number of violations regarding assumptions; hence, we proceeded with the analysis [67]. We selected a significance level of α=0.05.

**Fig 4:**
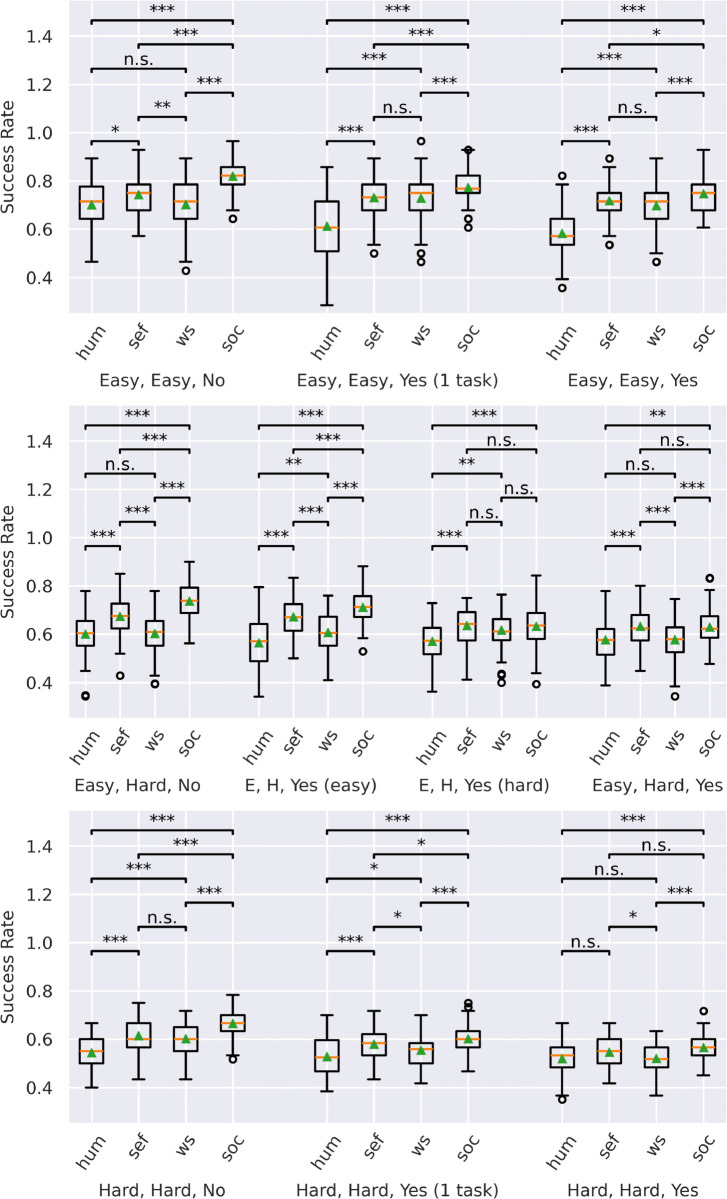
Mean success rate for each block and agent types. Mean SR for each of the ten blocks, as well as for each agent or baseline (hum = humans, sef = switch-every-frame, nS = no-SoC agent, soc = SoC agent). The median SR is represented by a line, while the mean SR is depicted as a triangle. Stars indicate significant differences in SRs.

Each of the ANOVAs performed across ten blocks yielded significant results, with all p-values falling below 0.01: p≤0.01 (easy_easy_no: F = 39.21; easy_easy_yes-in-one-task: F = 32.98; easy_easy_yes: F = 44.61; easy_hard_no: F = 65.42; easy_hard_yes-in-easy-task: F = 51.36; easy_hard_yes-in-hard-task: F = 8.97; easy_hard_yes: F = 14.13; hard_hard_no: F = 49.89; hard_hard_yes-in-one-task: F = 17.68; hard_hard_yes: F = 11.07). Therefore, we calculated the significance levels for each post-hoc two-tailed t-test. To account for multiple comparisons, p-values were adjusted with the Holm method [68]. The results for each block, including the performance and significance levels of each agent and the corresponding baselines, are presented in Fig 4.

The agent equipped with an SoC (*SoC* agent) exhibits markedly superior performance across nearly all scenarios compared to the *no-SoC* agent that does not utilize SoC, as well as both baselines *switch-every-frame* and *human* participants. An exception occurs in the situation where one task is easy, and the other one is hard, plus input noise affecting the harder task. In this particular case, the *no-SoC* agent and the baseline *switch-every-frame* achieve performance levels akin to those of the *SoC* agent. In addition, the baseline method *switch-every-frame* demonstrates an SR comparable to that of the *SoC* agent across scenarios involving both easy and hard tasks with input noise, as well as in instances characterized by two hard tasks with input noise. Furthermore, the overall SR declines under conditions of input noise at the same level of difficulty. This degradation in performance is also observed when varying the difficulty levels while maintaining consistent input noise.

These results raise the compelling question: What accounts for the superior performance of the *SoC* agent? To investigate this, we undertook a comprehensive analysis of task-switching behavior across all blocks and among the various agents and baselines involved in the study.

### Task-switching behavior

The overall performance factor for multitasking switching is represented by the mean of the SR for both subtasks. However, an SR of 0.5 could occur if one subtask is completed perfectly while the other is entirely uncompleted. Thus, it is necessary to analyze the **performance factors for each separate task**. As illustrated in S1 Table, all agents and baseline models achieve a minimum SR of 0.46 at each task. This observation indicates that the trained meta-agents act according to a balanced policy, where neither task is neglected in favor of the other within the multitasking scenario.

To further substantiate the effectiveness of task management, we analyze the **ratio of engagement in each task**, which quantifies the proportion of steps dedicated by the agent, baseline, or human to a specific task relative to the total number of steps. We only present the ratio of task one because the ratio of engagement for task two can be derived by subtracting the ratio of task one from 1. The mean ratios for each agent type and block are detailed in Table 3.

**Table 3:** Mean ratio of being in task one per episode.

Diff 1	Diff 2	I.n.	Hum	Sef	No-soc	Soc
easy	easy	no	0.49 (± 0.06)	0.50 (± 0.00)	0.47 (± 0.14)	0.50 (± 0.04)
		one	0.50 (± 0.06)	0.50 (± 0.00)	0.43 (± 0.11)	0.59 (± 0.05)
		yes	0.48 (± 0.08)	0.50 (± 0.00)	0.32 (± 0.08)	0.50 (± 0.06)
easy	hard	no	30.42 (± 0.07)	0.50 (± 0.00)	0.49 (± 0.12)	10.40 (± 0.05)
		easy	00.45 (± 0.07)	0.50 (± 0.00)	0.32 (± 0.10)	0.49 (± 0.06)
		hard	0.44 (± 0.07)	0.50 (± 0.00)	0.47 (± 0.10)	0.24 (± 0.05)
		yes	10.40 (± 0.08)	0.50 (± 0.00)	0.44 (± 0.11)	0.34 (± 0.06)
hard	hard	no	20.51 (± 0.10)	0.50 (± 0.00)	0.41 (± 0.06)	0.50 (± 0.07)
		one	0.50 (± 0.08)	0.50 (± 0.00)	60.57 (± 0.09)	0.65 (± 0.06)
		yes	0.49 (± 0.09)	0.50 (± 0.00)	70.54 (± 0.10)	0.49 (± 0.09)

Mean ratios of engagement in task one per episode for each block and agent type (Hum = humans, Sef = switch-every-frame, No-soc = no-SoC agent, Soc = SoC agent). The term “Diff 1” refers to the difficulty level associated with task one, while “Diff 2” pertains to the difficulty level of task two. The abbreviation “I.n.” denotes the input noise level corresponding to each specific block configuration. The ratio of engagement for task two can be derived by subtracting the ratio of task one from 1. Note that in the easy, easy and hard, hard blocks, when only one task includes input noise, task one is affected by the input noise.

Unsurprisingly, the baseline *switch-every-frame* maintains a consistent ratio of 0.5 for both tasks, reflecting equal involvement. Similarly, *humans* typically divide their time evenly between both tasks, with the highest difference in ratios for block easy_hard with input noise. In this block, humans spend only 40% of their time on the easy task while spending 60% of their time on the hard task. In contrast, the *no-SoC* agent demonstrates no noticeable pattern in task engagement, whereas the *SoC* agent shows a tendency to prioritize the more complex task. For example, in scenarios where the task difficulty is the same but one involves input noise, the ratios engaging in the task with input noise are higher: easy_easy with input noise in one task yields a ratio of 0.59 ± 0.05 in the task with input noise; hard_hard with input noise in one task results in 0.65 ± 0.06 in the task with input noise. In circumstances where one task is easy while the other is hard, the *SoC* agent predominantly engages with the more challenging task. Therefore, the ratios of being in task one, which is the easier task, are only 0.40 ± 0.05 for the scenario lacking input noise, and 0.34 ± 0.06 when both tasks exhibit input noise. This effect is strengthened when adding input noise only in the harder task, resulting in a ratio of only 0.24 ± 0.05 in the easy task. This observation changes when the easy task has input noise involved, whereas the hard task does not, resulting in ratios close to 0.5. Additionally, when both tasks present similar difficulties and input noise levels, the ratios are close to 0.5 once again.

To analyze the evolution of performances among various agents and baseline conditions, we evaluate the **number of switches** in Table 4.

**Table 4:** Mean number of switches per episode.

Diff1	Diff2	I.n.	Hum	Sef	No-soc	Soc
easy	easy	no	23.20 (± 9.41)	94.00 (± 0.00)	21.55 (± 4.80)	55.05 (± 4.81)
		one	23.11 (± 8.59)	94.00 (± 0.00)	24.89 (± 5.15)	45.92 (± 4.70)
		yes	22.72 (± 9.86)	94.00 (± 0.00)	21.23 (± 4.31)	39.44 (± 4.64)
easy	hard	no	20.02 (± 7.68)	94.00 (± 0.00)	22.14 (± 5.69)	43.99 (± 4.89)
		easy	19.28 (± 8.59)	94.00 (± 0.00)	20.85 (± 6.30)	39.37 (± 4.16)
		hard	19.83 (± 8.34)	94.00 (± 0.00)	16.23 (± 3.77)	25.55 (± 3.55)
		yes	18.46 (± 7.00)	94.00 (± 0.00)	14.37 (± 3.34)	27.48 (± 4.09)
hard	hard	no	18.33 (± 7.90)	94.00 (± 0.00)	31.82 (± 4.61)	37.92 (± 4.16)
		one	16.07 (± 7.10)	94.00 (± 0.00)	28.09 (± 5.15)	27.34 (± 3.70)
		yes	15.63 (± 5.90)	94.00 (± 0.00)	27.91 (± 5.09)	23.65 (± 3.07)

Mean number of switches per episode recorded for each block and agent type (hum = humans, sef = switch-every-frame, no-soc = no-SoC agent, soc = SoC agent). The term “Diff 1” refers to the difficulty level associated with task one, while “Diff 2” pertains to the difficulty level of task two. The abbreviation “I.n.” denotes the input noise level corresponding to each specific block configuration. Note that in the easy, easy and hard, hard blocks, when only one task includes input noise, task one is affected by this input noise.

We observe that the baseline *switch-every-frame* switches every five frames in over 470 steps, resulting in a total of 94 switches per episode. In contrast, both agents with and without an SoC, along with human participants, demonstrate significantly lower switching frequencies. Specifically, *human* participants exhibit an average switch range of 15.63 to 23.20 switches per trial, accompanied by a notable standard deviation of up to 9.86. For humans, a negative correlation is evident between the task difficulty and the number of switches, indicating that as tasks become more challenging, the frequency of switches decreases. Both the introduction of input noise and the transition from easy to hard task conditions correspond with this decline in switching behavior. The *no-SoC* agent shows no discernible trend, reflecting a range of 14.37 ± 3.34 switches in the easy_hard with input noise in both tasks block and 31.82 ± 4.61 switches in the hard_hard without input noise block. In the analysis of the three levels of difficulty combinations, where both tasks are at an easy level, one task is at an easy level while the other is at a hard level, or both tasks are at a hard level, the *SoC* agent demonstrates a consistent reduction in the number of switches when input noise is introduced, mirroring the observed behavior of human participants. However, unlike humans, the agent does not exhibit a clear downward trend across the three difficulty levels. Specifically, the number of switches observed in the more difficult level combination without input noise is higher than that in the easier level combination where input noise is present. Generally, the number of switches recorded for the *SoC* agent is quite higher than that of the *no-SoC* agent and *human* subjects. The *SoC* agent displays a range from 23.65 ± 3.07 switches in the hard_hard with input noise condition to 55.05 ± 4.81 switches in the easy_easy without input noise condition. Yet, despite this increased switching frequency, it remains considerably less than the 94 switches observed in the baseline, which switches as often as possible.

We further aimed at analyzing the **duration until a task switch** is performed per block and agent. However, the data exhibits significant noise, characterized by large standard deviations and numerous outliers, which precludes any meaningful analysis.

It is noteworthy that the baseline *switch-every-frame* underperforms with regard to mean SR, relative to the agent employing an SoC across the majority of blocks. This observation suggests that the situation in which the agent transitions between tasks is crucial. To explore this further, we compare the circumstances surrounding task-switching with those of non-switching situations in terms of the **number of visible asteroids**. The mean number of asteroids visible in switching and non-switching situations is shown in S2 Table. In total, there are 94 potential task-switching situations per episode observed over 100 episodes, yielding 9,400 potential task-switching instances. For the *no-SoC* agent, we were unable to find a discernible trend regarding the number of visible asteroids in task-switching versus non-switching situations. Note that non-switching situations do not exist for the baseline *switch-every-frame*. Still, further analysis reveals that both *human* participants and the *SoC* agent consistently tend to switch tasks when a smaller number of asteroids is visible (as compared to non-switching situations). However, it is evident that the frequency of switches is often less than half of the number of possible switching situations, resulting in a substantially greater number of non-switching instances. High standard deviations further complicate the quantitative analysis of object counts. Consequently, a qualitative evaluation of these metrics is not feasible.

**Fig 5:**
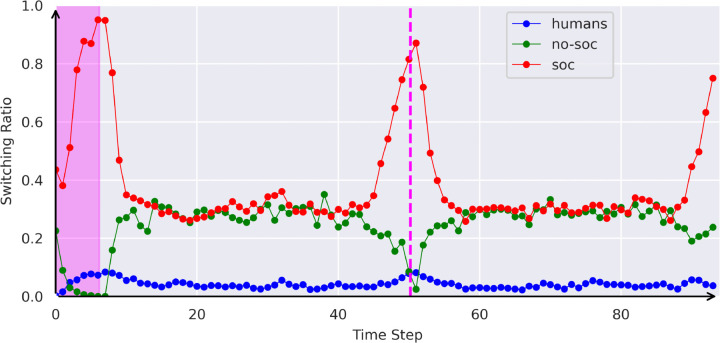
Switching ratio over trial. Switching ratios of humans, the no-SoC agent, and the SoC agent over the course of 94 time steps, where it is possible to switch. The pink areas mark the phases where no asteroids are visible. Note that we exclude the baseline that switches every frame.

To further elucidate the specific role of the need for control component in task-switching, we incorporated phases without asteroids both at the beginning and midpoint of each trial. According to the definition of NfC, the need for control will be minimal during these phases, as no action control is required. We then evaluate the **ratio of switches per timestep**. We present the switch ratio for each time step and agent across all blocks in Fig 5. Because the baseline *switch-every-frame* has a ratio of 1 in every time step, we do not consider it in the figure.

Observations revealed that while the *SoC* agent initiates switches in every frame when no asteroids are visible, the *no-SoC* agent refrains from switching entirely under similar conditions. There is no substantial evidence indicating that *human* subjects exhibit increased switching in the absence of asteroids.

## Discussion

The results of our evaluation studies indicate that a hierarchical RL agent utilizing an SoC exhibits superior performance compared to agents lacking an SoC or following a fixed strategy, such as switching tasks every possible frame, and can even outperform human participants. Analyzing the overall success rate of the different agents and humans, we observed that both the agents and the *switch-every-frame* baseline outdo *human* participants. Several factors may contribute to this finding. Firstly, while our analysis of human data supports the feasibility of switching tasks after five frames (approximately half a second), it is probable that humans cannot maintain this switching efficiency consistently throughout each trial, a capability that agents, including the aforementioned baseline, have. Secondly, previous studies have indicated that humans experience switching costs during task transitions [11,12,41,69]. This phenomenon may lead to a resumption lag, resulting in a time loss before they can effectively engage with a subtask after switching. Conversely, agents and the baseline *switch-every-frame* are able to execute actions directly following a switch. Further, a general exhaustion effect resulting from the completion of 20 trials of a similar task may impact human performance. Thirdly, it is essential to consider that the data representing human performance is an average across all participants for each block. Additionally, human participants were limited to two trials per block, resulting in only 46 data points per block for 23 participants, thus rendering the data noisier and necessitating cautious interpretation of human performance.

The superior performance of the *SoC* agent compared to the *no-SoC* agent, baseline conditions, and human participants is notably more pronounced in easier tasks. For instance, the *SoC* agent significantly outperforms all other evaluated conditions in the easy_easy block, across all input noise scenarios, where both tasks are relatively straightforward. Furthermore, the *SoC* agent consistently demonstrates better performance in blocks characterized by no input noise or only input noise in one task, such as easy_hard_no, easy_hard_yes-in-easy-task, hard_hard_no, and hard_hard_yes-in-one-task. Conversely, in blocks that present more challenging configurations, the *SoC* agent only surpasses the *no-SoC* agent and the baseline agent in specific instances. We hence conclude that the mechanisms underlying the SoC can be more distinctly differentiated in easier tasks, where smaller sections signal a need for control and no input noise is present reducing prediction errors. In these scenarios with lower control requirements, the SoC effectively facilitates the learning of successful task-switching policies. In contrast, in more difficult tasks including input noise, the prediction errors and need for control estimates are consistently higher, resulting in a permanently lower SoC. It seems that this circumstance impairs the agent’s ability to learn how to differentiate between situational demands effectively.

When comparing the time ratios of being in control of task one vs. two per episode, we see that the incorporation of SoC in a hierarchical RL agent enhances its ability to discern and prioritize the more challenging task, directing greater focus accordingly. In contrast, the agent without SoC and human participants exhibit problems in categorizing tasks based on difficulty. It seems that the ability to effectively prioritize different tasks is critical, as well as the decision on when to switch. Looking at the number of switches, it is not sufficient to just switch as fast as possible (like the baseline agent); sometimes omitting a switch may enhance overall performance. The key question is, of course, when to switch best, and whether an AI agent can learn this policy autonomously. The performance of the SoC agent indicates that this can not be based only on merely observable parameters (e.g., number of asteroids), but that components of an SoC, such as prediction errors and need for control, are helpful, which inherently rest on an agent’s ability to predict and assess the effects of its actions in different environmental states. Our analyses of switching- and non-switching-situations suggest that both *humans* and the *SoC* agent engage in task transitions in environments where they exhibit a sense of safety and a diminished need for control. This is indicated by the reduced number of asteroids visible during task-switching. This switching in situations where fewer asteroids are displayed and the need for control is lower seems to result in higher performance. This is proven by the results of the switching ratio over trial. The pronounced switching frequency observed in the *SoC* agent when no asteroids are present indeed suggests that it has effectively learned to execute switching actions in contexts where no need for control is present.

To sum up, our analyses indicate that the implementation of an SoC in agents correlates with better learning and enhanced performance in multitasking scenarios. This improvement arises because, unlike the *no-SoC* agent, the baseline model *switch-every-frame*, and *humans*, the *SoC* agent acquires an ability to concentrate on the more challenging task that necessitates greater control. Consistent with human behavior, it learns that increased switching is advantageous in situations with fewer asteroids while also recognizing the need to minimize switching in more demanding situations. Nonetheless, the *SoC* agent outperforms the baseline *switch-every-frame* in the majority of blocks, suggesting that remaining engaged in a current task can be more beneficial in specific contexts for effectively coping with upcoming situations with complex action control challenges.

### Limitations

As mentioned above, comparison to humans is partly difficult because they played fewer evaluation trials than the agents, and have higher standard deviations in some measurements. While humans may have lower switching costs in our study, compared to other multitasking scenarios, because we choose the same tasks for both subtasks, they are still present. Additionally, having to control the same task twice in a multitasking scenario rarely represents real-world experience. Similarly, collecting and analyzing human data in a laboratory setting can reduce individuals’ autonomy over the task and diminish their intrinsic motivation to engage in it. In the future, different subtasks and domains should be tested and evaluated. Also, the current mathematical modeling of a situational SoC is limited primarily to prediction errors and the need for control component. Subsequent investigations should examine additional “postdictive” SoC cues, such as the feedback obtained during or after task execution. Another important aspect to consider would be a model of an SoC for the meta-agent itself, which may capture the agent’s perception of its overall control of the multitasking situation in following a specific switching policy.

## Conclusion

In addressing the complexities associated with serial multitasking scenarios involving continuous tasks in RL, we demonstrated that RL agents can greatly benefit from the incorporation of cognitive control mechanisms, specifically the situational SoC. To facilitate this, we developed a specialized multitasking environment designed to enable precise manipulation of factors that influence the SoC. We have proposed and implemented a hierarchical RL agent architecture tailored to the multitasking scenario in this environment. In addition, we devised a mathematical model of the situational aspects of an SoC, which we successfully integrated within the RL agents. Our evaluation involved a comparative analysis of this implementation and two other configurations: one without the SoC and a baseline that switches tasks as frequently as possible. Furthermore, we included comparisons with the performance of human participants. The results indicated that RL agents employing an SoC outperformed both agents without an SoC, the baseline switch-every-frame, and human participants across nearly all experimental conditions. We can thus conclude that incorporating a model-based account of cognitive control mechanisms in AI agents such as autonomous robots or mobile vehicles that must learn how to act in complex, possibly uncertain environments is a promising approach and, at least, helps to identify and elucidate suitable behavioral strategies.

Looking ahead, we aim to extend our experimental setup to applications in various domains and different subtasks within a domain. One compelling opportunity is the integration of an SoC in robots operating in real-world environments. For instance, an SoC could significantly reduce the likelihood of robots falling, particularly by mitigating the increased prediction errors encountered when navigating unfamiliar terrains and diverse ground conditions. Additionally, we envision the development of more sophisticated agent configurations and learning regimes, such as an agent integrating an SoC while also receiving external rewards on the task currently being performed.

## Supporting information

S1 Table Mean success rates per task and agent. The table displays the mean success rates (SR) for tasks one (1) and two (2) for each block and agent type. The term “Diff 1” refers to the difficulty level associated with task one, while “Diff 2” pertains to the difficulty level of task two. The abbreviation “I.n.” denotes the input noise level corresponding to each specific block configuration. Note that in the easy, easy and hard, hard blocks, when only one task includes input noise, task one is affected by this input noise.
(PDF)

S2 Table Mean number of asteroids visible in switching and non-switching situations. The table displays the mean number of asteroids visible in switching (swi. sit.) and non-switching situations (non-swi. sit.) for each block and agent. The agent switch-every-frame is skipped because it has no non-switching situations. The term “Diff 1” refers to the difficulty level associated with task one, while “Diff 2” pertains to the difficulty level of task two. The abbreviation “I.n.” denotes the input noise level corresponding to each specific block configuration. Note that in the easy, easy and hard, hard blocks, when only one task includes input noise, task one is affected by this input noise.
(PDF)

## Declaration of generative AI and AI-assisted technologies

During the preparation of this work, we utilized ChatGPT 4o (OpenAI) and Grammarly to enhance the readability of individual sentences. In addition, GitHub Copilot assisted by providing suggestions for the detailed formulations, commenting, and formatting of source code that was used for the evaluation of human studies and the implementation of agents. The agent implementation involves approximately 2200 lines of code, which includes the environment class, wrapper classes, classes for various agent versions (baseline and agent with a sense of control), as well as code dedicated to hyperparameter tuning. The evaluation of human studies is carried out in a Jupyter notebook that comprises 2854 lines of code. Although the researchers developed this code, GitHub Copilot was consistently utilized for suggestions on code completion, primarily for variable names and boilerplate code related to basic operations, such as retrieving values from data frames. We accepted some suggestions while rejecting others; however, none involved significant recommendations like entire functions, classes, or algorithms. All code developed with Copilot’s assistance has undergone manual verification, and test functions have been created manually to ensure code validity. GitHub Copilot was not involved in the creation of concepts, mathematical definitions, methodologies, or any considerations related to study design and evaluation. After using these tools, we reviewed and edited the content as necessary, taking full responsibility for the final manuscript and code. We declare that, to the best of our knowledge, the content is accurate and valid, there are no concerns about potential plagiarism, all relevant sources are cited, and all statements in the article reporting hypotheses, results, conclusions, limitations, and implications of the study represent the authors’ own ideas.
