# Supplementary material for: Giving AI agents a sense of control facilitates reinforcement learning in multitasking scenarios
Source: PLoS One. 2026 Feb 6;21(2):e0342305. doi: 10.1371/journal.pone.0342305 (PMC12880638; doi:10.1371/journal.pone.0342305)
Supplement: S1 Table — The table displays the mean success rates (SR) for tasks one (1) and two (2) for each block and agent type. The term “Diff 1” refers to the difficulty level associated with task one, while “Diff 2” pertains to the difficulty level of task two. The abbreviation “I.n.” denotes the input noise level corresponding to each specific block configuration. Note that in the easy, easy and hard, hard blocks, when only one task includes input noise, task one is affected by this input noise. (PDF) [file pone.0342305.s001.pdf]

			Humans		Switch-every-frame		No-soc-agent		Soc-agent	
Diff 1	Diff 2	I.n.	SR 1	SR 2	SR 1	SR 2	SR 1	SR 2	SR 1	SR 2
easy	easy	no	0.72 (± 0.12)	0.68 (± 0.16)	0.73 (± 0.11)	0.75 (± 0.11)	0.72 (± 0.15)	0.68 (± 0.18)	**0.83 (± 0.10)**	**0.81 (± 0.11)**
		one	0.64 (± 0.15)	0.58 (± 0.16)	0.71 (± 0.12)	**0.75 (± 0.11)**	0.71 (± 0.15)	**0.75 (± 0.13)**	**0.83 (± 0.10)**	0.72 (± 0.12)
		yes	0.57 (± 0.15)	0.59 (± 0.13)	0.71 (± 0.12)	0.72 (± 0.11)	0.64 (± 0.14)	**0.76 (± 0.11)**	**0.75 (± 0.14)**	0.75 (± 0.11)
easy	hard	no	0.62 (± 0.12)	0.58 (± 0.10)	0.73 (± 0.11)	0.61 (± 0.09)	0.67 (± 0.15)	0.53 (± 0.10)	**0.74 (± 0.12)**	**0.73 (± 0.08)**
		easy	0.59 (± 0.16)	0.52 (± 0.09)	0.73 (± 0.13)	0.61 (± 0.09)	0.59 (± 0.18)	0.62 (± 0.11)	**0.77 (± 0.12)**	**0.66 (± 0.09)**
		hard	0.62 (± 0.13)	0.53 (± 0.11)	0.73 (± 0.11)	0.53 (± 0.10)	**0.77 (± 0.14)**	0.46 (± 0.12)	0.55 (± 0.14)	**0.71 (± 0.09)**
		yes	0.53 (± 0.14)	0.62 (± 0.09)	**0.72 (± 0.12)**	0.64 (± 0.09)	0.70 (± 0.16)	0.46 (± 0.12)	0.60 (± 0.16)	**0.66 (± 0.09)**
hard	hard	no	0.53 (± 0.10)	0.56 (± 0.09)	0.61 (± 0.08)	0.61 (± 0.09)	0.57 (± 0.10)	0.63 (± 0.11)	**0.67 (± 0.10)**	**0.66 (± 0.09)**
		one	0.56 (± 0.11)	0.49 (± 0.12)	0.54 (± 0.09)	**0.61 (± 0.09)**	0.52 (± 0.11)	0.59 (± 0.11)	**0.67 (± 0.10)**	0.53 (± 0.11)
		yes	0.54 (± 0.09)	0.49 (± 0.11)	0.54 (± 0.10)	0.55 (± 0.09)	0.51 (± 0.11)	0.53 (± 0.11)	**0.57 (± 0.10)**	**0.56 (± 0.10)**
