# Supplementary material for: Giving AI agents a sense of control facilitates reinforcement learning in multitasking scenarios
Source: PLoS One. 2026 Feb 6;21(2):e0342305. doi: 10.1371/journal.pone.0342305 (PMC12880638; doi:10.1371/journal.pone.0342305)
Supplement: S2 Table — The table displays the mean number of asteroids visible in switching (swi. sit.) and non-switching situations (non-swi. sit.) for each block and agent. The agent switch-every-frame is skipped because it has no non-switching situations. The term “Diff 1” refers to the difficulty level associated with task one, while “Diff 2” pertains to the difficulty level of task two. The abbreviation “I.n.” denotes the input noise level corresponding to each specific block configuration. Note that in the easy, easy and hard, hard blocks, when only one task includes input noise, task one is affected by this input noise. (PDF) [file pone.0342305.s002.pdf]

			Humans						No-SoC-Agent						SoC-Agent					
			CA 1			CA 2			CA 1			CA 2			CA 1			CA 2		
Diff 1	Diff 2	IN	Swi. Sit.	Non-Swi. Sit.	Sig.	Swi. Sit.	Non-Swi. Sit.	Sig.	Swi. Sit.	Non-Swi. Sit.	Sig.	Swi. Sit.	Non-Swi. Sit.	Sig.	Swi. Sit.	Non-Swi. Sit.	Sig.	Swi. Sit.	Non-Swi. Sit.	Sig.
		no	**0.58 (± 0.74)**	**0.95 (± 0.84)**	***	**0.61 (± 0.83)**	**1.06 (± 1.00)**	***	**0.73 (± 0.74)**	**0.98 (± 1.07)**	***	**0.90 (± 0.85)**	**0.72 (± 0.97)**	***	**0.58 (± 0.87)**	**1.59 (± 0.83)**	***	**0.55 (± 0.82)**	**1.58 (± 0.89)**	***
easy	easy	one	**0.49 (± 0.80)**	**1.05 (± 1.10)**	***	**0.48 (± 0.74)**	**1.02 (± 1.02)**	***	**0.86 (± 0.85)**	**0.79 (± 0.99)**	*	**0.97 (± 0.91)**	**0.73 (± 0.96)**	***	**0.39 (± 0.74)**	**1.46 (± 0.89)**	***	**0.54 (± 0.82)**	**1.64 (± 0.89)**	***
		yes	**0.44 (± 0.70)**	**1.01 (± 0.94)**	***	**0.41 (± 0.80)**	**1.07 (± 1.14)**	***	**0.70 (± 0.79)**	**1.31 (± 1.04)**	***	**1.09 (± 0.80)**	**0.58 (± 0.94)**	***	**0.36 (± 0.73)**	**1.46 (± 0.87)**	***	**0.40 (± 0.75)**	**1.43 (± 0.90)**	***
		no	**0.58 (± 0.80)**	**1.12 (± 1.08)**	***	**1.11 (± 1.43)**	**2.12 (± 1.68)**	***	**0.95 (± 0.92)**	**0.87 (± 0.99)**	*	**2.25 (± 1.74)**	**1.51 (± 1.27)**	***	**0.56 (± 0.88)**	**1.55 (± 0.80)**	***	**1.35 (± 1.63)**	**2.35 (± 1.25)**	***
easy	hard	easy	**0.50 (± 0.78)**	**1.11 (± 1.05)**	***	**1.00 (± 1.39)**	**2.09 (± 1.69)**	***	**0.66 (± 0.83)**	**1.41 (± 0.98)**	***	**2.63 (± 1.61)**	**1.29 (± 1.30)**	***	**0.41 (± 0.76)**	**1.46 (± 0.89)**	***	**1.32 (± 1.62)**	**2.35 (± 1.29)**	***
		hard	**0.51 (± 0.78)**	**1.09 (± 1.07)**	***	**1.12 (± 1.46)**	**2.16 (± 1.70)**	***	**0.22 (± 0.51)**	**1.43 (± 0.91)**	***	**2.35 (± 1.57)**	**1.08 (± 1.22)**	***	**0.63 (± 0.90)**	**1.55 (± 0.91)**	***	**1.26 (± 1.43)**	**2.06 (± 1.55)**	***
		yes	**0.41 (± 0.65)**	**1.00 (± 0.93)**	***	**1.02 (± 1.33)**	**2.05 (± 1.63)**	***	**0.19 (± 0.57)**	**1.46 (± 0.90)**	***	**2.50 (± 1.54)**	**1.13 (± 1.24)**	***	**0.43 (± 0.78)**	**1.46 (± 0.89)**	***	**1.10 (± 1.43)**	**2.19 (± 1.51)**	***
		no	**1.27 (± 1.39)**	**2.09 (± 1.48)**	***	**1.29 (± 1.42)**	**2.06 (± 1.59)**	***	1.96 (± 1.66)	1.90 (± 1.13)	ns	**1.98 (± 1.59)**	**1.45 (± 1.46)**	***	**1.29 (± 1.57)**	**2.37 (± 1.28)**	***	**1.26 (± 1.59)**	**2.36 (± 1.24)**	***
hard	hard	one	**1.15 (± 1.36)**	**2.04 (± 1.54)**	***	**1.22 (± 1.42)**	**2.02 (± 1.51)**	***	**2.26 (± 1.58)**	**1.22 (± 1.23)**	***	1.94 (± 1.70)	2.02 (± 1.24)	ns	**1.19 (± 1.48)**	**2.20 (± 1.52)**	***	**1.31 (± 1.59)**	**2.29 (± 1.24)**	***
		yes	**1.31 (± 1.34)**	**2.12 (± 1.48)**	***	**1.07 (± 1.34)**	**1.96 (± 1.56)**	***	**2.12 (± 1.62)**	**1.38 (± 1.31)**	***	**2.07 (± 1.66)**	**1.71 (± 1.29)**	***	**1.24 (± 1.48)**	**2.23 (± 1.49)**	***	**1.16 (± 1.41)**	**2.19 (± 1.53)**	***
