# Supplementary material for: Giving AI agents a sense of control facilitates reinforcement learning in multitasking scenarios
Source: PLoS One. 2026 Feb 6;21(2):e0342305. doi: 10.1371/journal.pone.0342305 (PMC12880638; doi:10.1371/journal.pone.0342305)
Supplement: Attachment — Submitted filename: Response to Reviewers.pdf [file pone.0342305.s003.pdf]

Response Letter for the reviewers of the manuscript “Giving AI agents a sense of control facilitates reinforcement learning in multitasking scenarios”

General Response:

Thank you for your thorough review of our manuscript. Your constructive feedback has genuinely strengthened our manuscript, and we appreciate the care and time you took in evaluating our work. Please find our detailed responses to the issues raised, along with the corresponding changes in the manuscript, in the section below. In the revised manuscript, changes from the previous version are marked using the \hl{} and \colorbox{} commands in the TeX file and highlighted in the PDF. Moreover, we have corrected the Data Availability link to ensure it points directly to the Open Science Framework (OSF) repository, rather than directing to it via doi.org. The updated link now provides direct access to our study's underlying data set on OSF, which meets the standards outlined by PLOS.

Reviewer #1:

Point 1:
1. Some sections (particularly the mathematical formulation in Modeling sense of control, Eqs. 7–12) are overly detailed for the main text. Suggestion: Move detailed derivations or step-by-step formulations to the Supplementary Materials and keep only the conceptual explanation in the main text.
Response: We appreciate your input and understand the rationale behind your proposal. However, since the supplementary material is provided as a separate PDF, it poses challenges for incorporating references that are used in these sections effectively, as it cannot directly link to the reference list in the main paper. Additionally, the sections in question are integral to the core narrative and findings of the paper, and thus, we believe they are best suited to remain within the main text.
To address your concerns, we have reduced the length of the section and simplified the formulas where possible. In detail, the formula for the distance calculation utilized in the prediction error formula has been removed and directly integrated into the calculation of the prediction error. Additionally, the equations for determining the next predicted state and associated reward have been consolidated into a single formula. Variable names have been abbreviated for clarity, for example, 'e' is used in place of 'env', 'm' instead of 'fm', the spaceship name has been eliminated from the x-coordinate, and the variables 'no_action' and 'input noise' have been removed. We also streamlined the formula for transitioning between states (Eq. 2 and Eq. 3), simplified the calculation of Euclidean distance by using absolute values instead of square roots and exponents, and simplified the need for control calculation by explaining the lower bounds in the accompanying text and removing them from the formula. Furthermore, we have enhanced the modeling section with a textual explanation and a relevant reference regarding minmax normalization. Minor formal adjustments include the modification of the delta

symbol in Equation 4 and moving the sentences after Equation 5 to before it to prevent a paragraph consisting of a single sentence.

Point 2:
2. The images are of low resolution, limiting the reader's ability to read. Fig.4 in particular is unreadable.
Response: Thank you for pointing this out. We have taken the necessary steps to improve the image quality and included higher resolution images to ensure clarity and enhance the overall presentation of our findings.

3. Minor Editorial Points
Point 3.1: Review the abbreviations for English words like Fig and Eq. As far as I know, it is usually Fig. 1 instead of Fig 1 as it is now. Same for the abbreviation Eq.
Response: Although it is more common in scientific papers to use a period to reference figures and equations, the current format without a period (e.g., "Fig 1" instead of "Fig. 1") is in accordance with the specific guidelines provided by the journal (see https://journals.plos.org/plosone/s/figures (In-text citations)). These guidelines require the use of abbreviations without a period.

Point 3.2: Correct small grammatical issues (e.g., “i.e. optimized” → “i.e., optimized”).
Response: Thank you for pointing out the grammatical issues in our paper. We have made the necessary corrections to address these issues and improve the clarity and quality of the manuscript. Below is a summary of the changes made:

- Added Commas:
    - Page 2: “Consider for example changing” → “Consider, for example, changing”
    - Page 4: “e.g. using a Likert scale” → “e.g., using a Likert scale”
    - Page 8: “whereas in the hard condition there are 30 asteroids” → “whereas in the hard condition, there are 30 asteroids”
    - Page 10: “i.e. optimized” → “i.e., optimized”
    - Page 14: “yet have not been substantiated in others [45] such that the specific array” → “yet have not been substantiated in others [45], such that the specific array”
    - Page 17: “optimal policy, i.e. given by” → “optimal policy, i.e., given by”
    - Page 22: “Scilab-RL/tree/v1.0” → “Scilab-RL/tree/v1.0.”
    - Page 25: “i.e. collection of all asteroids” → “i.e., collection of all asteroids”
    - Page 26: “where both subtasks are in an easy condition and no input noise is present” → “where both subtasks are in an easy condition, and no input noise is present”
    - Page 27: “Note that in the block where one task is easy and the other is hard” → “Note that in the block where one task is easy, and the other is hard”
    - Page 28: “An exception occurs in the situation where one task is easy and the other one is hard” → “An exception occurs in the situation where one task is easy, and the other one is hard”

    - Page 32: “Switching ratios of humans, the no-SoC agent, and the SoC agent over the course of 94 time steps where it is possible to switch” → “Switching ratios of humans, the no-SoC agent, and the SoC agent over the course of 94 time steps, where it is possible to switch”
    - Page 32: “or following a fixed strategy such as switching tasks every possible frame” → “or following a fixed strategy, such as switching tasks every possible frame”
    - Page 33: “ The key question is of course when to switch best” → “ The key question is, of course, when to switch best”
    - Page 34: “e.g. number of asteroids” → “e.g., number of asteroids”
    - Page 34: “ SoC such as prediction errors and need for control are helpful” → “SoC, such as prediction errors and need for control, are helpful”
    - Page 37: “student research assistants Zoe Klinger, Michel Wagemann, Dennis Reinhardt, and Laurin Gräsner for” → “student research assistants, Zoe Klinger, Michel Wagemann, Dennis Reinhardt, and Laurin Gräsner, for”
- “subagent” → “sub-agent”
- Page 2 + 7: “multitask” → “multitasking”
- Page 17: “subenvironment” → “sub-environment”
- Page 22: “received approval by the ethics committee of Bielefeld University” → “received approval from the ethics committee of Bielefeld University”
- Page 25 + 26 + 29 + 30: Upper Case in Table 1 and bold plus/minus symbol
- Page 26: “The first baseline *switch-every-frame* consists of an agent …. The second baseline are *human* …” → “The first baseline *switch-every-frame* is an agent …. The second baseline consists of *human* …”
- Page 29: “This observation changes when the easy task has input noise involved, whereas the hard task has not” → “This observation changes when the easy task has input noise involved, whereas the hard task does not”
- Page 33: “Looking at the number of switches, It” → “Looking at the number of switches, it”
- Page 34: “SoC agent indicates that this can not only base in merely observable parameters” → “SoC agent indicates that this can not be based only on merely observable parameters”
- Page 34: “Our analyses of switching- and non-switching-situations suggests” → “Our analyses of switching- and non-switching-situations suggest ”
- Page 34: “and the need for control is lower, seems to result in” → “and the need for control is lower seems to result in”
- Page 34: “coping with up-coming situations” → “coping with upcoming situations”

Point 3.3: Check consistency in section capitalization and formatting.
Response: According to the submission guidelines provided by the journal (https://journals.plos.org/plosone/s/submission-guidelines), ”Titles should be written in sentence case (only the first word of the text, proper nouns, and genus names are capitalized”. So, for example, in the manuscript, the title "Giving AI agents a sense of control facilitates

reinforcement learning in multitasking scenarios" follows this guideline, with "Giving" as the first word capitalized and "AI" as a proper noun. Similarly, "Collect Asteroids" is treated as a proper noun. Therefore, the section title "Subtasks - Collect Asteroids" capitalizes "Subtasks" as the first word, and "Collect Asteroids" as a proper noun. We have thoroughly reviewed all section titles for consistency in capitalization and formatting and found them to be in compliance with the journal's guidelines, so no changes were made. Please let us know if there are any specific instances you would like us to review further.

Reviewer Sarah D. Matthews, MPH, MS, PhD:

Point 1:
I bristled a bit at line 835 "human participants tend to perform worse...", there are other factors involved in human behavior (e.g. intrinsic motivation, empowerment) that are not addressed in the study design and in my humble opinion, this phrasing doesn't sit well. Maybe because I don't necessarily agree with the performance claims as written in the paper. But to their credit the authors address this somewhat in their discussion section and I do prefer the rephrasing in lines 927-929.
Response: We appreciate your perspective on the phrasing and understand the importance of considering factors like intrinsic motivation and empowerment in human behavior. We have revised the statement from “we observed that *human* participants tend to perform worse than both the agents and the *switch-every-frame* baseline” to “we observed that both the agents and the *switch-every-frame* baseline outdo *human* participants” to better reflect the nuances involved. Furthermore, we included an additional sentence in the Limitations section to acknowledge the absence of certain factors in the study design, such as intrinsic motivation and empowerment (see line 907-909: Similarly, collecting and analyzing human data in a laboratory setting can reduce individuals' autonomy over the task and diminish their intrinsic motivation to engage in it.).

Point 2:
I know outside the study, but I enjoyed reading the declaration information and how generative AI was used creatively in the study design.
Response: Thank you for your kind words. We are pleased to hear that you appreciated the declaration information on generative AI usage in the study design.

In addition to addressing the reviewer's comments, we have also implemented some of our own changes to further enhance the manuscript. The following changes were made:

- We have updated the DOI format to "https://doi.org/" so that it is possible to click on it in the PDF file.
- We have added a reference to Puterman in the context of POMDP and have corrected the observation transition probability.

- We have fixed the missing “+1” in line 545
- The Table S1 in Supporting Information has been reformatted to include colors, to be centered on the page, and to upper-case formatting.
- Table S2 in Supporting Information is now centered and formatted in upper case. We have also updated the content to display the total number of asteroids instead of their share.
- Figure 5: We have revised the figure to depict the switching ratio over trials across all blocks, rather than focusing on a specific block, to provide a more comprehensive analysis.
