# Supplementary material for: Giving AI agents a sense of control facilitates reinforcement learning in multitasking scenarios
Source: PLoS One. 2026 Feb 6;21(2):e0342305. doi: 10.1371/journal.pone.0342305 (PMC12880638; doi:10.1371/journal.pone.0342305)
Supplement: Peer review history

# Peer review: Decision Letter 0

**DOI:** 10.1371/journal.pone.0342305.r001

**Authors:** Tomislav Jagušt

8 Dec 2025

PONE-D-25-41872Giving AI agents a sense of control facilitates reinforcement learning in multitasking scenariosPLOS One

Dear Dr. Österdiekhoff,

Thank you for submitting your manuscript to PLOS ONE. After careful consideration, we feel that it has merit but does not fully meet PLOS ONE’s publication criteria as it currently stands. Therefore, we invite you to submit a revised version of the manuscript that addresses the points raised during the review process.

==============================

Both reviewers evaluated the manuscript positively, but also suggested several points for further improvement. Please read their comments carefully and address each point in your revision. In particular, please ensure that all requested clarifications, adjustments, and figure enhancements are incorporated. We look forward to receiving your revised version and response to reviewers.

==============================

Please submit your revised manuscript by Jan 22 2026 11:59PM. If you will need more time than this to complete your revisions, please reply to this message or contact the journal office at plosone@plos.org. When you're ready to submit your revision, log on to https://www.editorialmanager.com/pone/ and select the 'Submissions Needing Revision' folder to locate your manuscript file.

Please include the following items when submitting your revised manuscript:

- A rebuttal letter that responds to each point raised by the academic editor and reviewer(s). You should upload this letter as a separate file labeled 'Response to Reviewers'.
- A marked-up copy of your manuscript that highlights changes made to the original version. You should upload this as a separate file labeled 'Revised Manuscript with Track Changes'.
- An unmarked version of your revised paper without tracked changes. You should upload this as a separate file labeled 'Manuscript'.

If you would like to make changes to your financial disclosure, please include your updated statement in your cover letter. Guidelines for resubmitting your figure files are available below the reviewer comments at the end of this letter.

If applicable, we recommend that you deposit your laboratory protocols in protocols.io to enhance the reproducibility of your results. Protocols.io assigns your protocol its own identifier (DOI) so that it can be cited independently in the future. For instructions see: https://journals.plos.org/plosone/s/submission-guidelines#loc-laboratory-protocols. Additionally, PLOS ONE offers an option for publishing peer-reviewed Lab Protocol articles, which describe protocols hosted on protocols.io. Read more information on sharing protocols at https://plos.org/protocols?utm_medium=editorial-email&utm_source=authorletters&utm_campaign=protocols.

We look forward to receiving your revised manuscript.

Kind regards,

Tomislav Jagušt

Academic Editor

PLOS One

Journal requirements:

When submitting your revision, we need you to address these additional requirements.

1. Please ensure that your manuscript meets PLOS ONE's style requirements, including those for file naming. The PLOS ONE style templates can be found at

https://journals.plos.org/plosone/s/file?id=wjVg/PLOSOne_formatting_sample_main_body.pdf and

https://journals.plos.org/plosone/s/file?id=ba62/PLOSOne_formatting_sample_title_authors_affiliations.pdf

2. Thank you for uploading your study's underlying data set. Unfortunately, the repository you have noted in your Data Availability statement does not qualify as an acceptable data repository according to PLOS's standards.

At this time, please upload the minimal data set necessary to replicate your study's findings to a stable, public repository (such as figshare or Dryad) and provide us with the relevant URLs, DOIs, or accession numbers that may be used to access these data. For a list of recommended repositories and additional information on PLOS standards for data deposition, please see https://journals.plos.org/plosone/s/recommended-repositories.

3. If the reviewer comments include a recommendation to cite specific previously published works, please review and evaluate these publications to determine whether they are relevant and should be cited. There is no requirement to cite these works unless the editor has indicated otherwise.

4. Please review your reference list to ensure that it is complete and correct. If you have cited papers that have been retracted, please include the rationale for doing so in the manuscript text, or remove these references and replace them with relevant current references. Any changes to the reference list should be mentioned in the rebuttal letter that accompanies your revised manuscript. If you need to cite a retracted article, indicate the article’s retracted status in the References list and also include a citation and full reference for the retraction notice.

Additional Editor Comments (if provided):

[Note: HTML markup is below. Please do not edit.]

Reviewers' comments:

Reviewer's Responses to Questions

**Comments to the Author**

1. Is the manuscript technically sound, and do the data support the conclusions?

The manuscript must describe a technically sound piece of scientific research with data that supports the conclusions. Experiments must have been conducted rigorously, with appropriate controls, replication, and sample sizes. The conclusions must be drawn appropriately based on the data presented.

Reviewer #1: Yes

Reviewer #2: Yes

**********

2. Has the statistical analysis been performed appropriately and rigorously?

Reviewer #1: Yes

Reviewer #2: Yes

**********

3. Have the authors made all data underlying the findings in their manuscript fully available?

The PLOS Data policy requires authors to make all data underlying the findings described in their manuscript fully available without restriction, with rare exception (please refer to the Data Availability Statement in the manuscript PDF file). The data should be provided as part of the manuscript or its supporting information, or deposited to a public repository. For example, in addition to summary statistics, the data points behind means, medians and variance measures should be available. If there are restrictions on publicly sharing data—e.g. participant privacy or use of data from a third party—those must be specified.

Reviewer #1: Yes

Reviewer #2: Yes

**********

4. Is the manuscript presented in an intelligible fashion and written in standard English?

PLOS ONE does not copyedit accepted manuscripts, so the language in submitted articles must be clear, correct, and unambiguous. Any typographical or grammatical errors should be corrected at revision, so please note any specific errors here.

Reviewer #1: Yes

Reviewer #2: Yes

**********

5. Review Comments to the Author

Please use the space provided to explain your answers to the questions above. You may also include additional comments for the author, including concerns about dual publication, research ethics, or publication ethics. (Please upload your review as an attachment if it exceeds 20,000 characters)

Reviewer #1: This paper presents an innovative approach to enhancing reinforcement learning (RL) agents by introducing a situational sense of control (SoC) mechanism inspired by human cognition. The SoC integrates two key components—prediction error and need for control—and is incorporated into a hierarchical RL framework with a meta-agent managing task-switching. The approach is evaluated in a multitasking environment (“Collect Asteroids” game) and compared to human participants. Results indicate that the inclusion of SoC improves task-switching behavior and overall performance, suggesting that cognitive-inspired mechanisms can enhance RL systems. Overall, the paper is well written, methodologically sound, and contributes a novel concept bridging cognitive psychology and artificial intelligence.

1. Some sections (particularly the mathematical formulation in Modeling sense of control, Eqs. 7–12) are overly detailed for the main text. Suggestion: Move detailed derivations or step-by-step formulations to the Supplementary Materials and keep only the conceptual explanation in the main text.

2. The images are of low resolution, limiting the reader's ability to read. Fig.4 in particular is unreadable.

3. Minor Editorial Points

3.1. Review the abbreviations for English words like Fig and Eq. As far as I know, it is usually Fig. 1 instead of Fig 1 as it is now. Same for the abbreviation Eq.

3.2. Correct small grammatical issues (e.g., “i.e. optimized” → “i.e., optimized”).

3.3. Check consistency in section capitalization and formatting.

Reviewer #2: The manuscript is well-written and easy to read. The authors provided some great real world examples to help readers outside of computer science to understand the technical aspects of the study (line 12,70-91,etc.). I bristled a bit at line 835 "human participants tend to perform worse...", there are other factors involved in human behavior (e.g. intrinsic motivation, empowerment) that are not addressed in the study design and in my humble opinion, this phrasing doesn't sit well. Maybe because I don't necessarily agree with the performance claims as written in the paper. But to their credit the authors address this somewhat in their discussion section and I do prefer the rephrasing in lines 927-929. I know outside the study, but I enjoyed reading the declaration information and how generative AI was used creatively in the study design.

**********

6. PLOS authors have the option to publish the peer review history of their article (what does this mean?). If published, this will include your full peer review and any attached files.

If you choose “no”, your identity will remain anonymous but your review may still be made public.

**Do you want your identity to be public for this peer review?** For information about this choice, including consent withdrawal, please see our Privacy Policy.

Reviewer #1: No

Reviewer #2: **Yes:** Sarah D. Matthews, MPH, MS, PhD

**********

[NOTE: If reviewer comments were submitted as an attachment file, they will be attached to this email and accessible via the submission site. Please log into your account, locate the manuscript record, and check for the action link "View Attachments". If this link does not appear, there are no attachment files.]

To ensure your figures meet our technical requirements, please review our figure guidelines: https://journals.plos.org/plosone/s/figures

You may also use PLOS’s free figure tool, NAAS, to help you prepare publication quality figures: https://journals.plos.org/plosone/s/figures#loc-tools-for-figure-preparation.

NAAS will assess whether your figures meet our technical requirements by comparing each figure against our figure specifications.

# Author comment: Author response to Decision Letter 1

**DOI:** 10.1371/journal.pone.0342305.r002

22 Dec 2025

Following information also given in the Response to Reviewer Letter

Response Letter for the reviewers of the manuscript

“Giving AI agents a sense of control facilitates reinforcement learning in multitasking scenarios”

General Response:

Thank you for your thorough review of our manuscript. Your constructive feedback has genuinely strengthened our manuscript, and we appreciate the care and time you took in evaluating our work. Please find our detailed responses to the issues raised, along with the corresponding changes in the manuscript, in the section below. In the revised manuscript, changes from the previous version are marked using the \hl{} and \colorbox{} commands in the TeX file and highlighted in the PDF. Moreover, we have corrected the Data Availability link to ensure it points directly to the Open Science Framework (OSF) repository, rather than directing to it via doi.org. The updated link now provides direct access to our study's underlying data set on OSF, which meets the standards outlined by PLOS.

Reviewer #1:

Point 1:

1. Some sections (particularly the mathematical formulation in Modeling sense of control, Eqs. 7–12) are overly detailed for the main text. Suggestion: Move detailed derivations or step-by-step formulations to the Supplementary Materials and keep only the conceptual explanation in the main text.

Response: We appreciate your input and understand the rationale behind your proposal. However, since the supplementary material is provided as a separate PDF, it poses challenges for incorporating references that are used in these sections effectively, as it cannot directly link to the reference list in the main paper. Additionally, the sections in question are integral to the core narrative and findings of the paper, and thus, we believe they are best suited to remain within the main text.

To address your concerns, we have reduced the length of the section and simplified the formulas where possible. In detail, the formula for the distance calculation utilized in the prediction error formula has been removed and directly integrated into the calculation of the prediction error. Additionally, the equations for determining the next predicted state and associated reward have been consolidated into a single formula. Variable names have been abbreviated for clarity, for example, 'e' is used in place of 'env', 'm' instead of 'fm', the spaceship name has been eliminated from the x-coordinate, and the variables 'no_action' and 'input noise' have been removed. We also streamlined the formula for transitioning between states (Eq. 2 and Eq. 3), simplified the calculation of Euclidean distance by using absolute values instead of square roots and exponents, and simplified the need for control calculation by explaining the lower bounds in the accompanying text and removing them from the formula. Furthermore, we have enhanced the modeling section with a textual explanation and a relevant reference regarding minmax normalization. Minor formal adjustments include the modification of the delta symbol in Equation 4 and moving the sentences after Equation 5 to before it to prevent a paragraph consisting of a single sentence.

Point 2:

2. The images are of low resolution, limiting the reader's ability to read. Fig.4 in particular is unreadable.

Response: Thank you for pointing this out. We have taken the necessary steps to improve the image quality and included higher resolution images to ensure clarity and enhance the overall presentation of our findings.

3. Minor Editorial Points

Point 3.1: Review the abbreviations for English words like Fig and Eq. As far as I know, it is usually Fig. 1 instead of Fig 1 as it is now. Same for the abbreviation Eq.

Response: Although it is more common in scientific papers to use a period to reference figures and equations, the current format without a period (e.g., "Fig 1" instead of "Fig. 1") is in accordance with the specific guidelines provided by the journal (see https://journals.plos.org/plosone/s/figures (In-text citations)). These guidelines require the use of abbreviations without a period.

Point 3.2: Correct small grammatical issues (e.g., “i.e. optimized” → “i.e., optimized”).

Response: Thank you for pointing out the grammatical issues in our paper. We have made the necessary corrections to address these issues and improve the clarity and quality of the manuscript. Below is a summary of the changes made:

Added Commas:

Page 2: “Consider for example changing” → “Consider, for example, changing”

Page 4: “e.g. using a Likert scale” → “e.g., using a Likert scale”

Page 8: “whereas in the hard condition there are 30 asteroids” → “whereas in the hard condition, there are 30 asteroids”

Page 10: “i.e. optimized” → “i.e., optimized”

Page 14: “yet have not been substantiated in others [45] such that the specific array” → “yet have not been substantiated in others [45], such that the specific array”

Page 17: “optimal policy, i.e. given by” → “optimal policy, i.e., given by”

Page 22: “Scilab-RL/tree/v1.0” → “Scilab-RL/tree/v1.0.”

Page 25: “i.e. collection of all asteroids” → “i.e., collection of all asteroids”

Page 26: “where both subtasks are in an easy condition and no input noise is present” → “where both subtasks are in an easy condition, and no input noise is present”

Page 27: “Note that in the block where one task is easy and the other is hard” → “Note that in the block where one task is easy, and the other is hard”

Page 28: “An exception occurs in the situation where one task is easy and the other one is hard” → “An exception occurs in the situation where one task is easy, and the other one is hard”

Page 32: “Switching ratios of humans, the no-SoC agent, and the SoC agent over the course of 94 time steps where it is possible to switch” → “Switching ratios of humans, the no-SoC agent, and the SoC agent over the course of 94 time steps, where it is possible to switch”

Page 32: “or following a fixed strategy such as switching tasks every possible frame” → “or following a fixed strategy, such as switching tasks every possible frame”

Page 33: “ The key question is of course when to switch best” → “ The key question is, of course, when to switch best”

Page 34: “e.g. number of asteroids” → “e.g., number of asteroids”

Page 34: “ SoC such as prediction errors and need for control are helpful” → “SoC, such as prediction errors and need for control, are helpful”

Page 37: “student research assistants Zoe Klinger, Michel Wagemann, Dennis Reinhardt, and Laurin Gräsner for” → “student research assistants, Zoe Klinger, Michel Wagemann, Dennis Reinhardt, and Laurin Gräsner, for”

“subagent” → “sub-agent”

Page 2 + 7: “multitask” → “multitasking”

Page 17: “subenvironment” → “sub-environment”

Page 22: “received approval by the ethics committee of Bielefeld University” → “received approval from the ethics committee of Bielefeld University”

Page 25 + 26 + 29 + 30: Upper Case in Table 1 and bold plus/minus symbol

Page 26: “The first baseline switch-every-frame consists of an agent …. The second baseline are human …” → “The first baseline switch-every-frame is an agent …. The second baseline consists of human …”

Page 29: “This observation changes when the easy task has input noise involved, whereas the hard task has not” → “This observation changes when the easy task has input noise involved, whereas the hard task does not”

Page 33: “Looking at the number of switches, It” → “Looking at the number of switches, it”

Page 34: “SoC agent indicates that this can not only base in merely observable parameters” → “SoC agent indicates that this can not be based only on merely observable parameters”

Page 34: “Our analyses of switching- and non-switching-situations suggests” → “Our analyses of switching- and non-switching-situations suggest ”

Page 34: “and the need for control is lower, seems to result in” → “and the need for control is lower seems to result in”

Page 34: “coping with up-coming situations” → “coping with upcoming situations”

Point 3.3: Check consistency in section capitalization and formatting.

Response: According to the submission guidelines provided by the journal (https://journals.plos.org/plosone/s/submission-guidelines), ”Titles should be written in sentence case (only the first word of the text, proper nouns, and genus names are capitalized”. So, for example, in the manuscript, the title "Giving AI agents a sense of control facilitates reinforcement learning in multitasking scenarios" follows this guideline, with "Giving" as the first word capitalized and "AI" as a proper noun. Similarly, "Collect Asteroids" is treated as a proper noun. Therefore, the section title "Subtasks - Collect Asteroids" capitalizes "Subtasks" as the first word, and "Collect Asteroids" as a proper noun. We have thoroughly reviewed all section titles for consistency in capitalization and formatting and found them to be in compliance with the journal's guidelines, so no changes were made. Please let us know if there are any specific instances you would like us to review further.

Reviewer Sarah D. Matthews, MPH, MS, PhD:

Point 1:

I bristled a bit at line 835 "human participants tend to perform worse...", there are other factors involved in human behavior (e.g. intrinsic motivation, empowerment) that are not addressed in the study design and in my humble opinion, this phrasing doesn't sit well. Maybe because I don't necessarily agree with the performance claims as written in the paper. But to their credit the authors address this somewhat in their discussion section and I do prefer the rephrasing in lines 927-929.

Response: We appreciate your perspective on the phrasing and understand the importance of considering factors like intrinsic motivation and empowerment in human behavior. We have revised the statement from “we observed that human participants tend to perform worse than both the agents and the switch-every-frame baseline” to “we observed that both the agents and the switch-every-frame baseline outdo human participants” to better reflect the nuances involved. Furthermore, we included an additional sentence in the Limitations section to acknowledge the absence of certain factors in the study design, such as intrinsic motivation and empowerment (see line 907-909: Similarly, collecting and analyzing human data in a laboratory setting can reduce individuals' autonomy over the task and diminish their intrinsic motivation to engage in it.).

Point 2:

I know outside the study, but I enjoyed reading the declaration information and how generative AI was used creatively in the study design.

Response: Thank you for your kind words. We are pleased to hear that you appreciated the declaration information on generative AI usage in the study design.

In addition to addressing the reviewer's comments, we have also implemented some of our own changes to further enhance the manuscript. The following changes were made:

We have updated the DOI format to "https://doi.org/" so that it is possible to click on it in the PDF file.

We have added a reference to Puterman in the context of POMDP and have corrected the observation transition probability.

We have fixed the missing “+1” in line 545

The Table S1 in Supporting Information has been reformatted to include colors, to be centered on the page, and to upper-case formatting.

Table S2 in Supporting Information is now centered and formatted in upper case. We have also updated the content to display the total number of asteroids instead of their share.

Figure 5: We have revised the figure to depict the switching ratio over trials across all blocks, rather than focusing on a specific block, to provide a more comprehensive analysis.

Attachment Submitted filename: Response to Reviewers.pdf

# Peer review: Decision Letter 1

**DOI:** 10.1371/journal.pone.0342305.r003

**Authors:** Tomislav Jagušt

20 Jan 2026

Giving AI agents a sense of control facilitates reinforcement learning in multitasking scenarios

PONE-D-25-41872R1

Dear Dr. Österdiekhoff,

We’re pleased to inform you that your manuscript has been judged scientifically suitable for publication and will be formally accepted for publication once it meets all outstanding technical requirements.

Within one week, you’ll receive an e-mail detailing the required amendments. When these have been addressed, you’ll receive a formal acceptance letter and your manuscript will be scheduled for publication.

An invoice will be generated when your article is formally accepted. Please note, if your institution has a publishing partnership with PLOS and your article meets the relevant criteria, all or part of your publication costs will be covered. Please make sure your user information is up-to-date by logging into Editorial Manager at Editorial Manager® and clicking the ‘Update My Information' link at the top of the page. For questions related to billing, please contact billing support.

If your institution or institutions have a press office, please notify them about your upcoming paper to help maximize its impact. If they’ll be preparing press materials, please inform our press team as soon as possible -- no later than 48 hours after receiving the formal acceptance. Your manuscript will remain under strict press embargo until 2 pm Eastern Time on the date of publication. For more information, please contact onepress@plos.org.

Kind regards,

Tomislav Jagušt

Academic Editor

PLOS One

Additional Editor Comments (optional):

Dear authors of the manuscript "Giving AI agents a sense of control facilitates reinforcement learning in multitasking scenarios",

I am pleased to inform you that the peer review process for your manuscript has been successfully completed, and the editorial decision is: Accept.

Thank you for choosing PLOS ONE for publishing your research results. We appreciate your contribution to the scientific community and hope you will consider submitting your future research to PLOS One as well. I also wish you a lot of success in your future academic and research career.

Best regards,

Tomislav Jagušt

Reviewers' comments:

Reviewer's Responses to Questions

**Comments to the Author**

1. If the authors have adequately addressed your comments raised in a previous round of review and you feel that this manuscript is now acceptable for publication, you may indicate that here to bypass the “Comments to the Author” section, enter your conflict of interest statement in the “Confidential to Editor” section, and submit your "Accept" recommendation.

Reviewer #1: All comments have been addressed

**********

2. Is the manuscript technically sound, and do the data support the conclusions?

The manuscript must describe a technically sound piece of scientific research with data that supports the conclusions. Experiments must have been conducted rigorously, with appropriate controls, replication, and sample sizes. The conclusions must be drawn appropriately based on the data presented.

Reviewer #1: Yes

**********

3. Has the statistical analysis been performed appropriately and rigorously?

Reviewer #1: Yes

**********

4. Have the authors made all data underlying the findings in their manuscript fully available?

The PLOS Data policy requires authors to make all data underlying the findings described in their manuscript fully available without restriction, with rare exception (please refer to the Data Availability Statement in the manuscript PDF file). The data should be provided as part of the manuscript or its supporting information, or deposited to a public repository. For example, in addition to summary statistics, the data points behind means, medians and variance measures should be available. If there are restrictions on publicly sharing data—e.g. participant privacy or use of data from a third party—those must be specified.

Reviewer #1: Yes

**********

5. Is the manuscript presented in an intelligible fashion and written in standard English?

PLOS ONE does not copyedit accepted manuscripts, so the language in submitted articles must be clear, correct, and unambiguous. Any typographical or grammatical errors should be corrected at revision, so please note any specific errors here.

Reviewer #1: Yes

**********

6. Review Comments to the Author

Please use the space provided to explain your answers to the questions above. You may also include additional comments for the author, including concerns about dual publication, research ethics, or publication ethics. (Please upload your review as an attachment if it exceeds 20,000 characters)

Reviewer #1: The authors addressed all my comments. I don't have any further comments on the paper. I think the manuscript is ready for publication.

**********

7. PLOS authors have the option to publish the peer review history of their article (what does this mean?). If published, this will include your full peer review and any attached files.

If you choose “no”, your identity will remain anonymous but your review may still be made public.

**Do you want your identity to be public for this peer review?** For information about this choice, including consent withdrawal, please see our Privacy Policy.

Reviewer #1: No

**********

# Editor report: Acceptance letter

**DOI:** 10.1371/journal.pone.0342305.r004

**Authors:** Tomislav Jagušt

PONE-D-25-41872R1

PLOS One

Dear Dr. Österdiekhoff,

I'm pleased to inform you that your manuscript has been deemed suitable for publication in PLOS One. Congratulations! Your manuscript is now being handed over to our production team.

At this stage, our production department will prepare your paper for publication. This includes ensuring the following:

* All references, tables, and figures are properly cited

* All relevant supporting information is included in the manuscript submission,

* There are no issues that prevent the paper from being properly typeset

You will receive further instructions from the production team, including instructions on how to review your proof when it is ready. Please keep in mind that we are working through a large volume of accepted articles, so please give us a few days to review your paper and let you know the next and final steps.

Lastly, if your institution or institutions have a press office, please let them know about your upcoming paper now to help maximize its impact. If they'll be preparing press materials, please inform our press team within the next 48 hours. Your manuscript will remain under strict press embargo until 2 pm Eastern Time on the date of publication. For more information, please contact onepress@plos.org.

You will receive an invoice from PLOS for your publication fee after your manuscript has reached the completed accept phase. If you receive an email requesting payment before acceptance or for any other service, this may be a phishing scheme. Learn how to identify phishing emails and protect your accounts at https://explore.plos.org/phishing.

If we can help with anything else, please email us at customercare@plos.org.

Thank you for submitting your work to PLOS ONE and supporting open access.

Kind regards,

PLOS ONE Editorial Office Staff

on behalf of

Dr. Tomislav Jagušt

Academic Editor

PLOS One
